# Extracellular Vesicles from *Ecklonia cava* and Phlorotannin Promote Rejuvenation in Aged Skin

**DOI:** 10.3390/md22050223

**Published:** 2024-05-15

**Authors:** Sosorburam Batsukh, Seyeon Oh, Ji Min Lee, Judy Hong Jin Joo, Kuk Hui Son, Kyunghee Byun

**Affiliations:** 1 Department of Anatomy & Cell Biology, College of Medicine, Gachon University, Incheon 21936, Republic of Korea; 2 Functional Cellular Networks Laboratory, Graduate School and Lee Gil Ya Cancer and Diabetes Institute, Gachon University, Incheon 21999, Republic of Korea; 3 Doctors Dermatologic Clinic, Gangdong Godeok, Seoul 05269, Republic of Korea; 4 Glassy Skin Clinic, Seoul 06010, Republic of Korea; 5 Department of Thoracic and Cardiovascular Surgery, Gachon University Gil Medical Center, Gachon University, Incheon 21565, Republic of Korea; 6 Department of Health Sciences and Technology, Gachon Advanced Institute for Health & Sciences and Technology (GAIHST), Gachon University, Incheon 21999, Republic of Korea

**Keywords:** *Ecklonia cava*, extracellular vesicles from *Ecklonia cava*, phlorotannin, skin rejuvenation

## Abstract

Plant-derived extracellular vesicles (EVs) elicit diverse biological effects, including promoting skin health. EVs isolated from *Ecklonia cava* (EV-EC) carry heat shock protein 70 (HSP70), which inhibits key regulators such as TNF-α, MAPKs, and NF-κB, consequently downregulating matrix metalloproteinases (MMPs). Aging exacerbates oxidative stress, upregulating MAPK and NF-κB signaling and worsening extracellular matrix degradation in the skin. *E. cava*-derived phlorotannin (PT) mitigates MAPK and NF-κB signaling. We evaluated the impact of EV-EC and PT on skin rejuvenation using an in vitro keratinocyte senescence model and an in vivo aged-mouse model. Western blotting confirmed the presence of HSP70 in EV-EC. Treatment with EV-EC and PT in senescent keratinocytes increased HSP70 expression and decreased the expression of TNF-α, MAPK, NF-κB, activator protein-1 (AP-1), and MMPs. Oxidative stress was also reduced. Sequential treatment with PT and EV-EC (PT/EV-EC) yielded more significant results compared to individual treatments. The administration of PT/EV-EC to the back skin of aged mice mirrored the in vitro findings, resulting in increased collagen fiber accumulation and improved elasticity in the aged skin. Therefore, PT/EV-EC holds promise in promoting skin rejuvenation by increasing HSP70 expression, decreasing the expression of MMPs, and reducing oxidative stress in aged skin.

## 1. Introduction

Extracellular vesicles (EVs) are particles with a lipid bilayer that are released from cells into the extracellular space [1,2,3]. Due to their diverse cargo, including nucleic acids, proteins, lipids, and metabolites, EVs influence neighboring cells upon release into the extracellular space [4]. Similar to mammalian cells, plant cells also secrete EVs [5], which demonstrate various biological effects, such as antifungal and antimicrobial activities [5,6,7]. Additionally, these EVs can transport small non-coding RNAs among cells, promoting proliferation or differentiation [8,9].

Several studies have explored the therapeutic potential of plant-derived EVs in cutaneous diseases. For example, EVs derived from wheatgrass juice enhance the proliferation and migration abilities of endothelial, epithelial, and fibroblast cells, thereby promoting wound healing [10]. Similarly, EVs derived from ginseng roots inhibit melanogenesis in ultraviolet B (UVB)-irradiated human melanocytes, leading to a reduction in senescence-associated-β-galactosidase (SA-β-Gal) activity, indicating anti-pigmentation and anti-senescence effects [11]. Seaweed-derived EVs, such as those from *Saccharina fusiforme* and *Codium fragile*, also demonstrate anti-pigmentation effects by reducing melanin synthesis in MNT-1 cells [12]. However, the precise mechanism underlying the anti-melanogenic effects of seaweed-derived EVs remains incompletely understood.

Plant EVs, such as those derived from *Salvia dominica*, carry a variety of chaperone proteins, including heat shock protein 70 (HSP70), HSP80, and HSP90 [13]. These HSPs are also found in EVs derived from grapes and citrus fruits [14,15,16]. HSP70 plays a crucial role in reducing cell death by inhibiting tumor necrosis factor (TNF)-induced apoptosis and suppressing the activity of mitogen-activated protein kinases (MAPKs) [17] and nuclear factor-kappa B (NF-κB), leading to the downregulation of matrix metalloproteinases (MMPs) [18].

During aging, increased skin oxidative stress leads to the upregulation of MAPK pathways and NF-κB signaling, contributing to extracellular matrix (ECM) degradation [19]. This process is associated with cosmetic concerns, such as sagging, wrinkles, and laxity, which are primarily attributed to heightened MMP expression. Elevated MMP levels lead to the degradation of collagen and elastin fibers within the ECM [20,21,22].

The epidermal basement membrane (BM) at the dermal–epidermal junction has a sheet-like structure and contributes to binding the dermis and epidermis together [23]. Key structural proteins of the primary BM include nidogen, perlecan, laminin, and collagen (types IV and VII) [23]. During aging, MMPs contribute to BM degradation [24,25], leading to BM thinning and reduced levels of BM proteins [26,27]. Laminin expression is decreased in areas with skin wrinkles [28], and senescent fibroblasts exhibit decreased collagen IV levels [29]. NF-κB is implicated in the reduction in collagen synthesis by upregulating NADPH oxidase (NOX), thereby intensifying oxidative stress [30]. This heightened oxidative stress, in turn, decreases transforming growth factor-beta (TGF-β) expression, ultimately resulting in decreased collagen synthesis [31].

Seaweeds contain phenolic compounds with diverse biological effects, including antioxidant, antibacterial, anti-allergic, and anti-inflammatory properties [32,33,34,35]. Dieckol, an *Ecklonia cava*-derived phlorotannin (referred to as PT in this study), exhibits protective effects against UVB-induced injury in human dermal fibroblasts by attenuating reactive oxygen species (ROS) and reducing MMP levels through the downregulation of NF-κB, activator protein 1 (AP-1), and MAPK pathways [36].

While seaweed-derived EVs are recognized for their ability to decrease melanin synthesis and PT exhibits protective effects on the skin, the potential of *E. cava*-derived EVs (referred to as EV-EC) to induce skin rejuvenation during aging remains unexplored. Our study aimed to investigate the impact of EV-EC on skin rejuvenation using an aged-mouse model. We hypothesized that EV-EC contains HSP70, which can downregulate the MAPK and NF-κB pathways by inhibiting TNF-α. This downregulation would consequently reduce MMP expression, thereby mitigating ECM and BM destruction. Additionally, the reduced NF-κB activation would lead to decreased NOX activity and oxidative stress, ultimately promoting increased TGF-β and collagen synthesis in aged skin. Furthermore, we explored the hypothesis that combined treatments of PT and EV-EC could enhance skin rejuvenation effects beyond those observed with singular treatments of EV-EC. Therefore, we also aimed to compare the skin rejuvenation effects among treatments of EV-EC and PT alone with those of combined EV-EC and PT treatments.

## 2. Results

### 2.1. Characterization of EV-EC and PT

**Figure 1:**
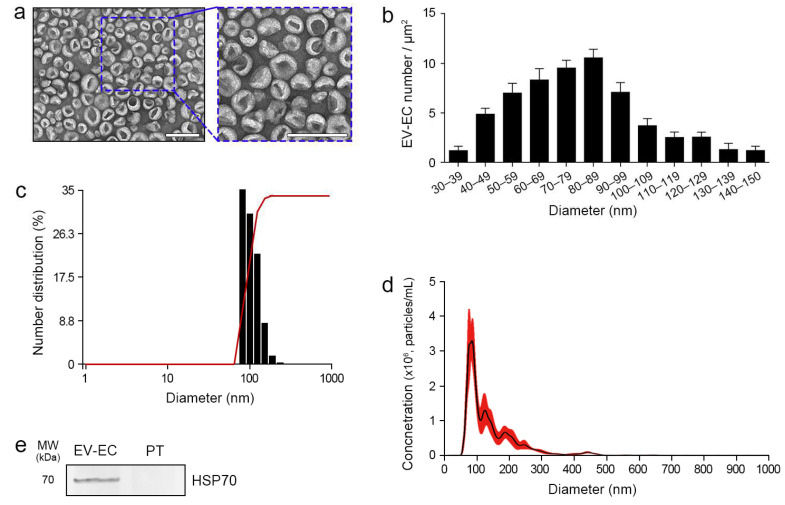
Characterization of EV-EC and detection of HSP70. (**a**) TEM images of the EV-EC. Blue dotted line indicates the magnified view (scale bar = 200 nm). (**b**) Quantification of EV-ECs per exosome diameter based on TEM images, with diameters ranging from 30 to 150 nm. (**c**) Number distribution of EV-ECs was provided by DLS analysis. (**d**) Particle size, mode of distribution, and concentration of EV-ECs were determined by NTA. (**e**) Western blot analysis confirmed the presence of HSP70 protein in EV-EC and PT. Data represent the mean ± SD of three independent experiments. DLS, dynamic light scattering; EV-EC, extracellular vesicles from *Ecklonia cava*; HSP70, heat shock protein 70; MW, molecular weight; NTA, nanoparticle-tracking analysis; PT, phlorotannin; SD, standard deviation; TEM, transmission electron microscopy.

Transmission electron microscopy (TEM) revealed the presence of intact, round EV-EC, predominantly ranging between 30 nm and 150 nm (Figure 1a,b). No evidence of EV aggregation was observed in TEM images. Dynamic light scattering (DLS) analysis and nanoparticle-tracking analysis (NTA) provided complementary insights into the size distribution of EV-EC particles. The mean particle size was determined to be 153.0 nm ± 39.9 nm by DLS and 137.6 ± 4.7 nm by NTA. Furthermore, NTA revealed a mode of distribution of particle size of 81.5 ± 3.1 nm, and the particle concentration was 3.68 × 10^10^ ± 6.40 × 10^9^ particles/mL/1g *E. cava* (Figure 1c,d and Table S1). The presence of HSP70 in EV-EC was confirmed via Western blotting. HSP70 was identified in EV-EC, while no detectable levels were observed in the PT solution (Figure 1e). Thus, these characterization analyses established the suitability of EV-EC for use in the subsequent experiments.

### 2.2. EV-EC and PT Decreased Senescence Marker Expression In Vitro

The cytotoxic effects of varying concentrations of EV-EC and PT (1–5000 μg/mL) on human epidermal keratinocytes (HEKn) were assessed after 24 h of incubation. No cytotoxicity was observed at concentrations up to 2000 μg/mL of EV-EC and PT (Figure S1).

To identify effective concentrations of EV-EC and PT that reduce cellular senescence, a senescence experiment was conducted using keratinocytes. Cellular senescence was induced by H_2_O_2_ treatment (Figure S2a), a commonly employed method used for simulating cell aging [37]. Ascorbic acid (AA), a well-known antioxidant that induces skin rejuvenation [38], was also used to treat H_2_O_2_-induced senescent (SnCs) keratinocytes for comparison with PT and EV-ECE treatment.

The expression of the senescence markers P21 and P16 was elevated upon H_2_O_2_ treatment. However, treatment with EV-EC at concentrations of 25–200 μg/mL resulted in a significant reduction in P21 and P16 expression (Figure S2b,c). Similarly, PT treatments at concentrations of 25–200 μg/mL led to a significant decrease in P21 and P16 expression in SnCs keratinocytes (Figure S2d,e). AA treatments at concentrations of 25–200 μM also led to a significant decrease in P21 and P16 in SnCs keratinocytes (Figure S2f,g). There was no significant difference among 50, 100, and 200 μg/mL PT or EV-EC treatments and 50, 100, and 200 μM AA treatments in reducing the expression of P21 and P16. Consequently, 50 μg/mL of PT and EV-EC was identified as a suitable concentration for subsequent experiments.

Given our hypothesis that PT enhances the skin rejuvenation effects of EV-EC, we investigated the optimal timing for PT treatment to maximize the impact of EV-EC. Three treatment methods were compared: (1) simultaneous treatment (EV-EC + PT), (2) pre-treatment with EV-EC followed by PT (EV-EC/PT), and (3) pre-treatment with PT followed by EV-EC (PT/EV-EC) (Figure S3a). The cell proliferation ratio was most significantly increased in the PT/EV-EC group (Figure S3b). Likewise, P21 and P16 expression was most significantly reduced in the PT/EV-EC group (Figure S3c,d). Consequently, the PT/EV-EC treatment method was selected for further experiments.

Taken together, EV-EC and PT effectively reduced the expression of senescence markers in human keratinocytes at 50 μg/mL following H_2_O_2_-induced senescence. The PT/EV-EC treatment exhibited the most substantial impact on cell proliferation and the greatest reduction in P21 and P16 expression, highlighting the synergistic anti-senescence effects of these agents.

### 2.3. EV-EC and PT Treatments Increased HSP70 Expression and Decreased Expression of TNF-α, MAPK, NF-κB, AP-1, and MMPs in Senescent Keratinocytes

HSP70 mRNA expression was significantly lower in SnCs keratinocytes compared to non-senescent (Non-SnCs) keratinocytes. Treatment with EV-EC, PT, PT/EV-EC, and AA increased HSP70 mRNA expression, with the most significant effect observed in the PT/EV-EC treatment group (Figure S4b). Conversely, SnCs keratinocytes exhibited higher TNF-α mRNA expression compared to Non-SnCs keratinocytes. Treatment with EV-EC, PT, PT/EV-EC, and AA decreased TNF-α mRNA expression, with the most significant effect observed in PT/EV-EC-treated SnCs keratinocytes (Figure S4c).

**Figure 2:**
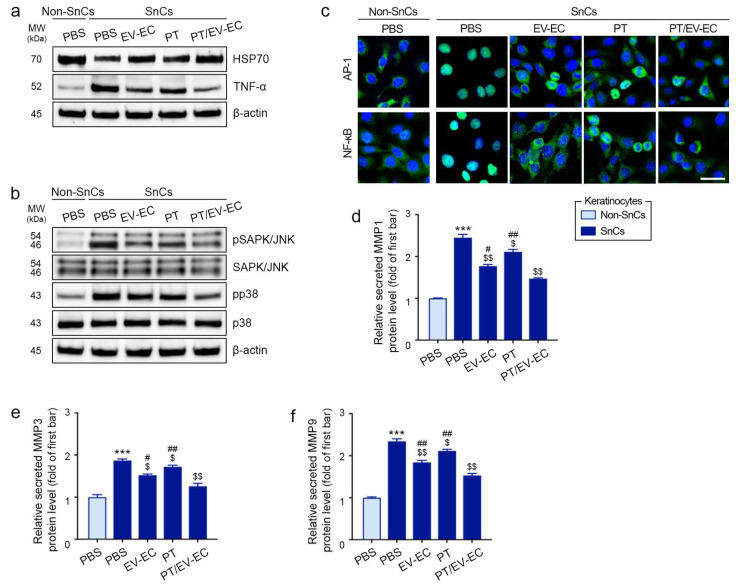
Modulation of HSP70, TNF-α, MAPK, AP-1, NF-κB, and MMP expression by EV-EC and PT in H_2_O_2_-induced SnCs keratinocytes. (**a**) Western blot analysis of HSP70, TNF-α, and β-actin levels in Non-SnCs and SnCs keratinocytes treated with PBS, EV-EC, PT, or PT/EV-EC. (**b**) Western blot analysis of pSAPK/JNK, total SAPK/JNK, pp38, total p38, and β-actin levels in Non-SnCs and SnCs keratinocytes. (**c**) ICC analysis of AP-1 and NF-κB expression (both green) in Non-SnCs and SnCs keratinocytes (nuclei: blue; scale bar = 30 μm). Quantitative data for (**a**–**c**) are presented in Figure S5. (**d**–**f**) ELISA evaluation of MMP1 (**d**), MMP3 (**e**), and MMP9 (**f**) protein levels in Non-SnCs and SnCs keratinocytes. Data represent the mean ± SD of three independent experiments. ***, *p* < 0.001, first bar vs. second bar; $, *p* < 0.05 and $$, *p* < 0.01, second bar vs. third, fourth, and fifth bars; #, *p* < 0.05 and ##, *p* < 0.01, fifth bar vs. third and fourth bars (Mann–Whitney U test). AP-1, activator protein-1; ELISA, enzyme-linked immunosorbent assay; ICC, immunocytochemistry; MMP, matrix metalloproteinase; NF-κB, nuclear factor kappa-light-chain-enhancer of activated B cells; Non-SnCs, non-senescent cells; PBS, phosphate-buffered saline; pp38, phosphorylated p38; pSAPK/JNK, phosphorylated SAPK/JNK; SAPK/JNK, stress-activated protein kinases/Jun-amino-terminal kinase; SnCs, senescent cells; TNF-α, tumor necrosis factor-alpha.

Subsequent experiments were conducted without including the AA treatment group, given its similar effects to EV-EC and PT in increasing HSP70 expression and decreasing TNF-α expression. The Western blotting results also showed that treatment with EV-EC, PT, and PT/EV-EC increased HSP70 expression, with the most significant effect observed in the PT/EV-EC treatment group (Figure 2a and Figure S5a). Conversely, SnCs keratinocytes exhibited higher TNF-α expression compared to Non-SnCs keratinocytes. Treatment with EV-EC, PT, and PT/EV-EC decreased TNF-α expression, with the most significant effect observed in PT/EV-EC-treated SnCs keratinocytes (Figure 2a and Figure S5b). 

The downstream effects of HSP70 and TNF-α, crucial for improving skin rejuvenation, were investigated. The evaluation of MAPKs (stress-activated protein kinase (SAPK)/jun amino-terminal kinase (JNK) and p38) in SnCs keratinocytes revealed increased ratios of phosphorylated SAPK/JNK to total SAPK/JNK (pSAPK/JNK/SAPK/JNK) and phosphorylated p38 to total p38 (pp38/p38) compared to Non-SnCs keratinocytes. Treatment with EV-EC, PT, and PT/EV-EC decreased both ratios, with the most significant effect observed in PT/EV-EC-treated SnCs keratinocytes (Figure 2b and Figure S5c,d).

The nuclear intensity of AP-1 and NF-κB staining was elevated in SnCs keratinocytes compared to Non-SnCs keratinocytes. Treatment with EV-EC, PT, and PT/EV-EC resulted in a significant decrease in the nuclear intensity of both AP-1 and NF-κB, with the most significant effect observed in the PT/EV-EC-treated SnCs keratinocytes (Figure 2c and Figure S5e,f).

The expression levels of MMP1, MMP3, and MMP9 were significantly higher in SnCs keratinocytes than in Non-SnCs keratinocytes. Treatment with EV-EC, PT, and PT/EV-EC led to a significant reduction in the expression of these MMPs, with the most significant effect observed in PT/EV-EC-treated SnCs keratinocytes (Figure 2d–f).

To evaluate whether increased HSP70 levels induced by PT/EV-EC resulted in decreased TNF-α, SAPK/JNK, p38, and NF-κB levels, we treated SnCs keratinocytes with an HSP70 inducer, geranylgeranylacetone (GGA). HSP70 expression, which was reduced in SnCs keratinocytes, was increased by PT/EV-EC or GGA treatment. Furthermore, when PT/EV-EC was administered to SnCs keratinocytes along with GGA, the expression of HSP70 was higher compared to treatments with PT/EV-EC or GGA alone (Figure S6a–c). The treatment of SnCs keratinocytes with PT/EVE or GGA led to a decrease in the expression of TNF-α, SAPK/JNK, p38, and NF-κB. Notably, the reduction in these factors was more pronounced with GGA than with PT/EV-EC alone (Figure S6a–h). These findings suggest that PT/EV-EC increases HSP70 expression, ultimately leading to the decreased expression of TNF-α, SAPK/JNK, p38, and NF-κB.

Subsequently, we assessed whether PT/EV-EC inhibited SAPK/JNK, p38, and AP-1 by treating SnCs keratinocytes with SAPK/JNK (SP600125) and p38 (SB203580) inhibitors (Figure S7). PT/EV-EC treatment decreased SAPK/JNK phosphorylation to levels similar to those achieved with the SAPK/JNK inhibitor (Figure S8a,b). Likewise, PT/EV-EC reduced p38 phosphorylation to levels comparable to those achieved with the p38 inhibitor (Figure S8b,c). Additionally, the expression of AP-1 was decreased by treatment with either of the SAPK/JNK or p38 inhibitors, as well as by PT/EV-EC (Figure S8e,f). Furthermore, the same treatments reduced the expression levels of MMP1, MMP3, and MMP9 (Figure S8g–i). These findings suggest that PT/EV-EC inhibits SAPK/JNK and p38, eventually leading to the decreased expression of AP-1, MMP1, MMP3, and MMP9.

In summary, treatment with EV-EC and PT ameliorated senescence-associated alterations in keratinocytes. This included the upregulation of HSP70 expression and the suppression of TNF-α, MAPKs (SAPK/JNK and p38), NF-κB, AP-1, and MMPs. These findings highlight the beneficial effects of EV-EC and PT in mitigating multiple facets of senescence in keratinocytes.

### 2.4. Attenuation of Oxidative Stress in Senescent Keratinocytes and Enhancement of TGF-β Expression by EV-EC and PT in Senescent Fibroblasts

**Figure 3:**
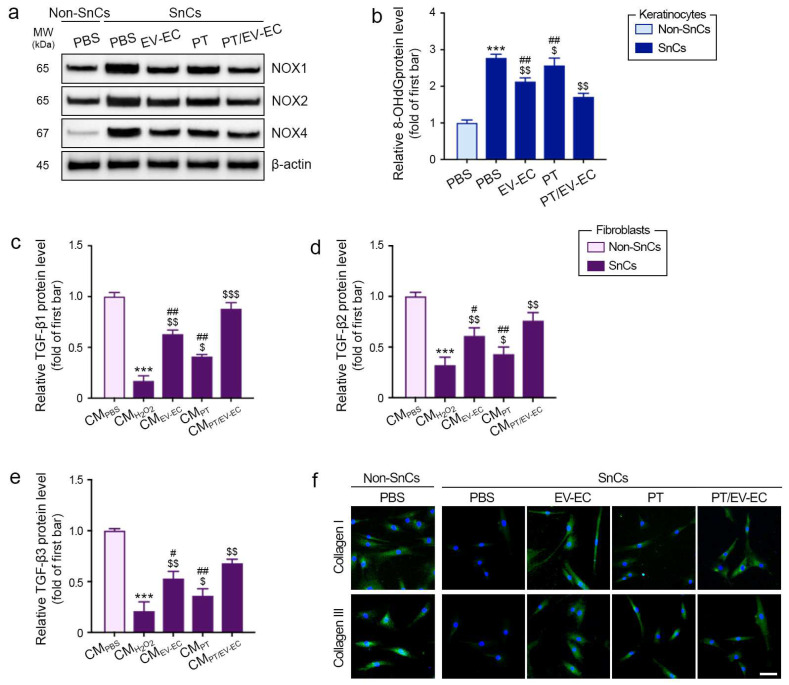
Reduction in reactive oxygen species (ROS) in SnCs keratinocytes by EV-EC and PT enhanced expression of TGF-β and collagen I and III in SnCs fibroblasts. (**a**) Western blot analysis of NOX1, NOX2, NOX4, and β-actin in Non-SnCs and SnCs keratinocytes treated with EV-EC, PT, and PT/EV-EC. Quantitative data are presented in Figure S9. (**b**) ELISA evaluation of 8-OhdG protein levels in Non-SnCs and SnCs keratinocytes. (**c**–**e**) ELISA assessment of TGF-β1 (**c**), TGF-β2 (**d**), and TGF-β3 I protein levels in Non-SnCs and SnCs fibroblasts treated with conditioned media (CM_PBS_, CM_H2O2_, CM_EV-EC_, CM_PT_, and CM_PT/EV-EC_). (**f**) ICC analysis of collagen I (upper) and collagen III (lower) expression (both green) in Non-SnCs and SnCs fibroblasts (nuclei: blue; scale bar = 30 μm). Quantitative data are presented in Figure S8. Data represent the mean ± SD of three independent experiments. ***, *p* < 0.001, first bar vs. second bar; $, *p* < 0.05 and $$, *p* < 0.01, second bar vs. third, fourth, and fifth bars; #, *p* < 0.05 and ##, *p* < 0.01, fifth bar vs. third and fourth bars (Mann–Whitney U test). 8-OHdG, 8-hydroxy-2′-deoxyguanosine; NOX, nicotinamide adenine dinucleotide phosphate oxidases; TGF-β, transforming growth factor-beta.

NOX enzymes play a pivotal role in ROS generation [39], with 8-hydroxy-2′-deoxyguanosine (8-OHdG) serving as a well-established marker for oxidative damage [40]. In SnCs keratinocytes, the levels of NOX1, NOX2, NOX4, and 8-OhdG were elevated compared to Non-SnCs keratinocytes. However, treatment with EV-EC, PT, and PT/EV-EC significantly decreased NOX1, NOX2, NOX4, and 8-OhdG expression, with the most pronounced effect observed with PT/EV-EC (Figure 3a,b, and Figure S9).

To further investigate these findings, we induced cellular senescence in fibroblasts by subjecting them to H_2_O_2_ treatment. Subsequently, conditioned media (CM) from keratinocytes were applied to Non-SnCs and SnCs fibroblasts. The CM was obtained from Non-SnCs keratinocytes (CM_PBS_) and SnCs keratinocytes treated with H_2_O_2_/PBS (CM_H2O2_), PT (CM_PT_), EV-EC (CM_EV-EC_), or PT/EV-EC (CM_PT/EV-EC_) (Figure S10).

We explored whether the reduction in oxidative stress in keratinocytes by PT or EV-EC could enhance TGF-β expression in SnCs fibroblasts through CM administration. The levels of TGF-β1, TGF-β2, and TGF-β3 in SnCs fibroblasts treated with CM_H2O2_ were lower than those in Non-SnCs fibroblasts treated with CM_PBS_. However, these levels increased in SnCs fibroblasts treated with CM_PT_, CM_EV-EC_, and CM_PT/EV-EC_, with the most significant enhancement observed in the SnCs fibroblasts treated with CM_PT/EV-EC_ (Figure 3c–e).

Similarly, the collagen I and III expression levels in SnCs fibroblasts treated with CM_H2O2_ were lower than those in Non-SnCs fibroblasts treated with CM_PBS_. Nevertheless, these levels increased in SnCs fibroblasts treated with CM_PT_, CM_EV-EC_, and CM_PT/EV-EC_, with the most significant enhancement observed in SnCs fibroblasts treated with CM_PT/EV-EC_ (Figure 3f and Figure S11).

We assessed whether PT/EV-EC could decrease the expression of NOXs and increase TGF-β expression by downregulating NF-κB. PT/EV-EC treatment resulted in decreased NF-κB expression comparable to that achieved with the NF-κB inhibitor SN50 in SnCs keratinocytes. Additionally, when PT/EV-EC was administered to SnCs keratinocytes, the level of 8-OhdG decreased to a similar extent to that observed with NF-κB inhibitor treatment. Furthermore, NOX1, NOX2, and NOX4 expression levels were reduced by PT/EV-EC treatment to a similar degree to that observed with NF-κB inhibitor treatment (Figures S12a and S13a–e).

CM_PBS_, CM_H2O2_, and CM_PT/EV_-_EC_, as well as CM from SnCs keratinocytes treated with the NF-κB inhibitor (CM_SN50_) or PT/EV-EC with the NF-κB inhibitor (CM_SN50PT/EV-EC_), were applied to Non-SnCs and SnCs fibroblasts. CM_PT/EV_-_EC_ increased TGF-β1, TGF-β2, and TGF-β3 expression to a similar extent to CM_SN50_ in the SnCs fibroblasts (Figures S12b and S13f–e).

In summary, EV-EC and PT reduced oxidative stress in SnCs keratinocytes (NOX1, NOX2, NOX4, and 8-OhdG) and promoted TGF-β expression in SnCs fibroblasts, enhancing collagen I and III levels. This highlights the potential interplay between keratinocytes and fibroblasts, demonstrating the rejuvenating impact of PT and EV-EC in response to senescence.

### 2.5. EV-EC and PT Increased HSP70 Expression and Decreased Expression of TNF-α, MAPK, NF-kB, AP-1, and MMPs in Aged Skin

To determine the optimal treatment dosages for skin, PT (0.5 and 1 mg/mL) and EV-EC (0.5 and 1 mg/mL) were dissolved in distilled water (DW). The skin of young (9-week-old) and aged (14-month-old) mice underwent injection via a microneedling system (MTS) with 200 μL of DW as the control or with 200 μL of the test mixtures (Figure S14a). Masson’s trichrome staining revealed reduced collagen fiber levels in the skin of aged mice compared to young mice. However, treatment with EV-EC or PT increased collagen fiber density, with the most pronounced effect observed at 1 mg/mL of both EV-EC and PT compared to 0.5 mg/mL (Figure S14b,c).

To assess collagen composition using Herovici’s stain, which differentiates between newly formed collagen (blue) and mature collagen (red) [41,42], we analyzed their densities in aged and young skin. Aged skin exhibited lower densities of both newly synthesized and mature collagen fibers compared to young skin. However, treatment with EV-EC or PT increased collagen density, with the 1 mg/mL treatments showing greater efficacy than the 0.5 mg/mL treatments (Figure S14b,d,e). Thus, further mouse experiments were conducted using the 1 mg/mL doses of PT and EV-EC.

To optimize the sequence of PT and EV-EC treatments for maximal collagen synthesis, young and aged mice were initially injected with 200 μL of DW in the dermis. After 7 days, another 200 μL of DW was administered. Aged mice were then subjected to three treatment protocols: (1) simultaneous injection with PT and EV-EC followed by DW injection after 7 days (PT + EV-EC group), (2) injection with EV-EC followed by PT injection after 7 days (EV-EC/PT group), and (3) injection with PT followed by EV-EC injection after 7 days (PT/EV-EC group) (Figure S15a). The PT/EV-EC group exhibited the highest collagen density, with both newly synthesized and mature collagen densities being the greatest in this group (Figure S15b–e).

Since PT/EV-EC was administered to the aged skin via an MTS in our study, we evaluated MTS-induced collagen synthesis more than the topical application. Topical application of PT/EV-EC significantly increased collagen fiber accumulation in the aged skin; however, treatment with PT/EV-EC by an MTS was more effective in increasing collagen fiber accumulation (Figure S16).

Subsequent mouse experiments focused on the PT/EV-EC treatment protocol. Therefore, in the primary mouse experiments, PT 1 mg/mL was injected into aged skin, followed by the injection of EV-EC 1 mg/mL after 7 days. These data were compared with young and aged control groups, where DW was injected at 7-day intervals (Figure S17).

**Figure 4:**
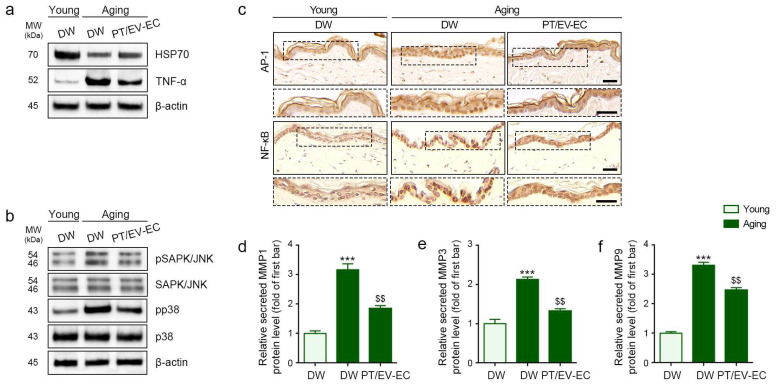
Modulation of HSP70, TNF-α, MAPK, AP-1, NF-κB, and MMPs expression by PT/EV-EC treatment in the skin of aged mice. (**a**) Western blot analysis of HSP70, TNF-α, and β-actin levels in the skin of young and aged mice. (**b**) Western blot analysis of pSAPK/JNK, total SAPK/JNK, pp38, total p38, and β-actin levels in the skin of young and aged mice. (**c**) IHC staining of AP-1 and NF-κB in the epidermis of young and aged mice (IHC signal: brown; nuclei: blue; scale bar = 80 μm). Quantitative data from (**a**–**c**) are presented in Figure S18. (**d**–**f**) ELISA assessment of MMP1, MMP3, and MMP9 in the skin of young and aged mice. Data represent the mean ± SD of three independent experiments. ***, *p* < 0.001, first bar vs. second bar; $$, *p* < 0.01, second bar vs. third bar (Mann–Whitney U test). AP-1, activator protein-1; DW, distilled water; IHC, immunohistochemistry.

Aged skin exhibited lower HSP70 expression and higher TNF-α expression compared to young skin. Notably, PT/EV-EC treatment effectively increased HSP70 expression, while concurrently reducing TNF-α expression (Figure 4a and Figure S18a,b). Additionally, the expression ratios of pSAPK/JNK/SAPK/JNK and pp38/p38 in aged skin were higher than those in young skin. These ratios were decreased by PT/EV-EC treatment in aged skin (Figure 4b and Figure S18c,d). Moreover, the nuclear intensity of AP-1 and NF-κB staining was elevated in aged skin compared to young skin, and this intensity was decreased by PT/EV-EC treatment in aged skin (Figure 4c and Figure S18e,f). Furthermore, the expression levels of MMP1, MMP3, and MMP9 in aged skin were higher than those in young skin but decreased with PT/EV-EC treatment (Figure 4d–f). These collective data support the rejuvenating effects of PT/EV-EC on aged skin by modulating key markers associated with inflammation and MMPs.

Taken together, these data indicate that PT/EV-EC treatment significantly improved collagen density and reduced the expression levels of inflammatory markers and MMPs in aged skin, suggesting its potential as a rejuvenating therapy for age-related skin changes.

### 2.6. EV-EC and PT Decreased Oxidative Stress and Increased TGF-β Expression in Aged Skin

**Figure 5:**
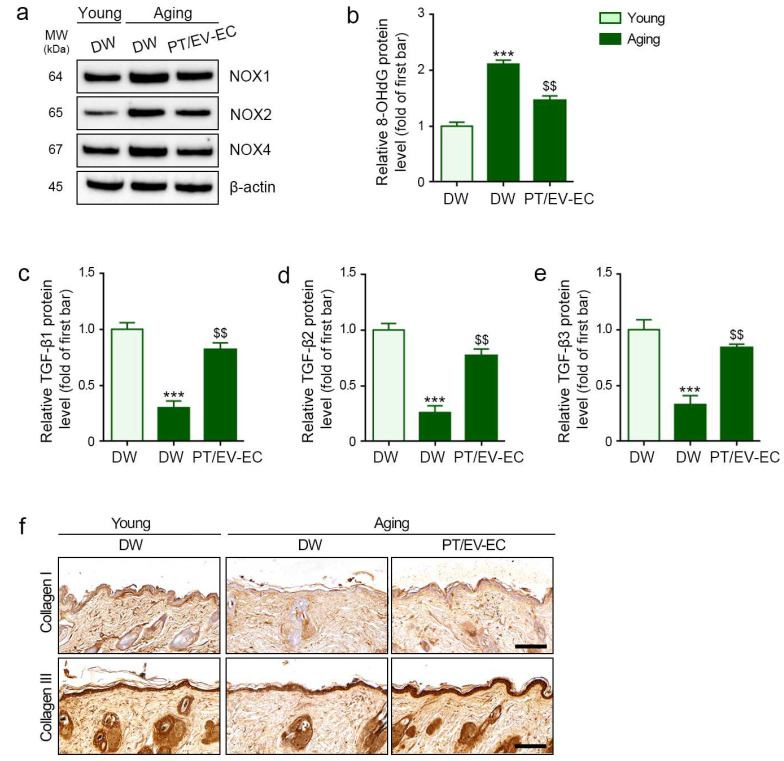
PT/EV-EC treatment reduced ROS levels and increased TGF-β1, TGF-β2, TGF-β3, and collagen I and III expression in the skin of aged mice. (**a**) Western blot analysis of NOX1, NOX2, NOX4, and β-actin in the skin of young and aged mice. (**b**–**e**) ELISA assessment of 8-OHdG, TGF-β1, TGF-β2, and TGF-β3 in the skin of young and aged mice. (**f**) IHC staining of collagen I (upper) and III (lower) in the dermis of young and aged mice (IHC signal: brown, nuclei: blue; scale bar = 100 μm). Quantitative data from (**a**,**f**) are presented in Figure S19. Data represent the mean ± SD of three independent experiments. ***, *p* < 0.001, first bar vs. second bar; $$, *p* < 0.01, second bar vs. third bar (Mann–Whitney U test).

In aged skin, elevated levels of NOX1, NOX2, and NOX4 indicated heightened oxidative stress compared to young skin. This increase was effectively mitigated by PT/EV-EC treatment (Figure 5a and Figure S19a–c). Simultaneously, the elevated levels of 8-OHdG in aged skin, indicative of oxidative damage, were significantly reduced by PT/EV-EC treatment (Figure 5b). This affirms the successful attenuation of oxidative stress by PT-EV-EC treatment.

Concomitantly, in aged skin, the expression of TGF-β1, TGF-β2, and TGF-β3, critical for tissue regeneration, was diminished. However, PT/EV-EC treatment led to a significant increase in the expression of these TGF-β isoforms (Figure 5c–e). The age-related decline in collagen I and III expression, with collagen being vital for skin structural integrity, was effectively countered by PT/EV-EC treatment (Figure 5f and Figure S19d,e), highlighting its impact on the structural integrity of aged skin.

### 2.7. PT/EV-EC Treatment Induced an Increase in BM-Related Proteins and Collagen Fiber in Aged Skin

**Figure 6:**
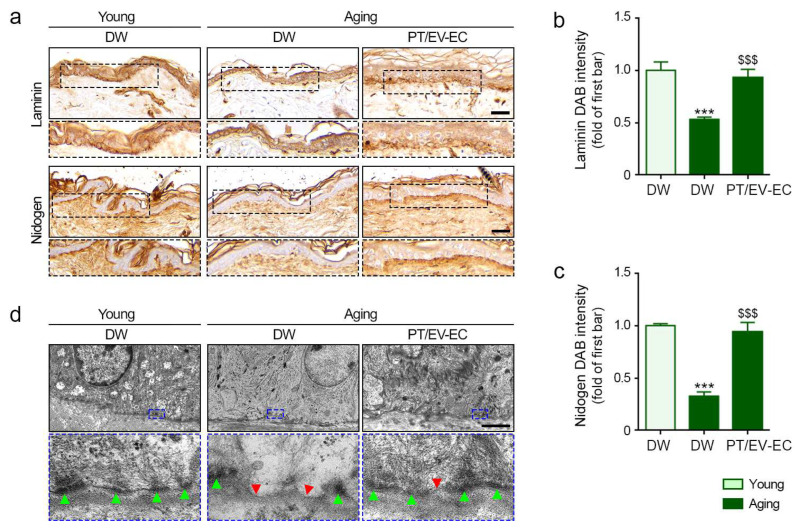
Basement membrane recovery in the skin of aged mice post EV-EC and PT treatment. (**a**) IHC staining of laminin (upper) and nidogen (lower) in the dermis of young and aged mice (IHC signal: brown, nuclei: blue; scale bar = 30 μm). (**b**–**c**) Quantification of laminin and nidogen signal intensities from the images in (**a**). (**d**) BM assessment via TEM imaging of young and aged skin (scale bar = 1 μm). Green marks represent hemidesmosomes, and red indicates lamina densa with disruptions or duplications. Data represent the mean ± SD of three independent experiments. ***, *p* < 0.001, first bar vs. second bar; $$$, *p* < 0.001, second bar vs. third bar (Mann–Whitney U test). BM, basement membrane.

Laminin and nidogen expression levels, significantly lower in aged skin compared to young skin, increased with PT/EV-EC treatment (Figure 6a–c). The BM, consisting of the lamina lucida, lamina densa, and lamina fibroreticularis [43], plays a crucial role in maintaining skin integrity. The lamina densa, with its sheet-like structure, exhibits disruptions or duplications during aging [26]. The lamina lucida is situated between the lamina densa and the epithelial layer [24]. Hemidesmosomes, electron-dense plaques responsible for attaching keratinocytes to the BM [24], were assessed using TEM.

In this study, TEM was utilized to assess hemidesmosomes and the lamina densa. The lamina densa of aged skin exhibited greater disruption compared to young skin, and the number of hemidesmosomes decreased in aged skin. PT/EV-EC treatment resulted in a reduction in lamina densa disruption and an increase in the number of hemidesmosomes in aged skin (Figure 6d), demonstrating its positive impact on skin structure.

**Figure 7:**
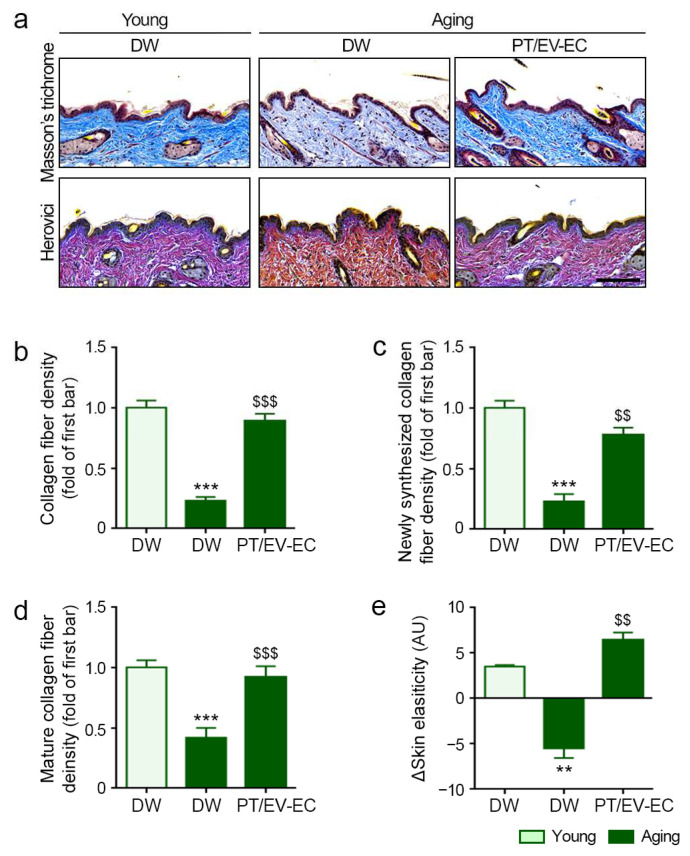
Enhanced rejuvenation by PT/EV-EC treatment in the skin of aged mice. (**a**) Masson’s trichrome staining (blue) depicting collagen fiber (upper) and Herovici’s staining of newly synthesized (blue) and mature (red) collagen fibers in skin samples from young and aged mice (scale bar = 100 µm). (**b**) Quantification of Masson’s trichrome staining from panel (**a**). (**c**,**d**) Quantification of Herovici’s staining from panel (**a**). (**e**) Skin elasticity before and 4 weeks after PT/EV-EC dermal injection in the skin of young and aged mice was measured using the API-100 instrument. Data represent the mean ± SD of three independent experiments. **, *p* < 0.01 and ***, *p* < 0.001, first bar vs. second bar; $$, *p* < 0.01 and $$$, *p* < 0.001, second bar vs. third bar (Mann–Whitney U test). AU, arbitrary unit.

Our findings indicate that PT/EV-EC treatment increases collagen I and III protein expression, influencing collagen accumulation in the skin. Collagen fiber density, assessed with Masson’s trichrome stain, was lower in aged skin than in young skin, but PT/EV-EC treatment significantly elevated collagen fiber density (Figure 7a,b). Additionally, the levels of newly formed collagen were reduced in aged skin but significantly increased following PT/EV-EC treatment. Similarly, mature collagen density, which declined in aged skin, showed significant improvement with PT/EV-EC (Figure 7a,c,d).

To comprehensively evaluate skin elasticity, we utilized API-100. Aged skin exhibited decreased elasticity, which was effectively reversed by PT/EV-EC treatment (Figure 7e). This evidence underscores the positive impact of PT/EV-EC on collagen synthesis and skin elasticity in the aging process.

Therefore, PT/EV-EC treatment shows promise in rejuvenating aging skin by enhancing BM proteins, promoting collagen synthesis, and improving structural integrity.

## 3. Discussion

Skin aging is a multifaceted process influenced by intrinsic and extrinsic factors. Intrinsic aging reflects chronological changes, characterized by the increased generation of ROS and cellular senescence [44]. Extrinsic factors, particularly UV radiation, exacerbate the skin aging process by accelerating cellular senescence [45]. SnCs cells, such as keratinocytes or fibroblasts, exhibit diminished proliferation abilities [46,47]. SnCs fibroblasts also demonstrate reduced production of type I procollagen, indicative of compromised TGF-β pathway signaling [48].

Given the crucial role of collagen fibers in maintaining skin elasticity [49,50], a decline in collagen leads to alterations in the skin matrix and the formation of wrinkles [49,50]. Type I collagen, primarily generated by dermal fibroblasts [51], is influenced by keratinocytes, which influence collagen synthesis or degradation through communication with fibroblasts [52,53,54]. UV radiation induces keratinocyte senescence, marked by elevated senescence markers such as p16, p21, and p53, increased SA-β-Gal activity, and enhanced ECM degradation [55,56]. These changes result in heightened ROS generation, subsequently increasing the expression of MAPKs, such as p38 and SAPK/JNK [55]. The activation of MAPK pathways upregulates AP-1, subsequently increasing MMP expression [57].

Efforts to counteract skin aging involve targeting key molecular pathways implicated in the aging process. Antioxidants, known for their ability to reduce ROS generation and inhibit the MAPK pathway, have shown promise in decreasing MMP activity and preventing collagen degradation [58]. Central to cellular protection, HSP70 inhibits MAPK pathways, reduces NOX activity, downregulates NF-κB [18,59], and enhances collagen synthesis [60].

To validate our extraction method for obtaining EV-EC, we initially identified EVs using TEM, confirming their presence with particle sizes ranging from 30 to 150 nm. Plant-derived EVs typically range from 30 to 1000 nm, with specific examples including orange (*Citrus aurantium*) at 105–396 nm, ginger (*Zingiber officinale*) at 125–250 nm, broccoli (*Brassica oleracea*) at 18–400 nm, and carrot (*Daucus carota*) at 100–1000 nm [61,62,63,64,65,66,67,68,69,70]. Additionally, EVs from seaweed species such as *S. fusiforme* and *C. fragile* have average particle sizes of 201.1 ± 15.04 nm and 148.6 ± 3.119 nm, respectively [12]. Notably, EV-EC, with a mean particle size of 137.6 ± 4.7 nm (Figure 1c), exhibited a particle size similar to those of plant- and seaweed-derived EVs.

The confirmation of HSP70’s presence in EV-EC underscores its potential involvement in molecular pathways linked to skin aging and collagen synthesis. Recognized for its skin-protective effects through MAPK pathway inhibition [36], the combination of PT with EV-EC holds promise for a synergistic impact on skin rejuvenation, potentially surpassing outcomes achievable through individual treatments with either EV-EC or PT.

In vitro studies using keratinocytes were conducted to assess cytotoxicity, revealing that neither EV-EC nor PT, even at concentrations as high as 2000 μg/mL, induced a decrease in cell viability. Shifting our focus to cellular senescence in keratinocytes, a significant reduction in the senescence markers P21 and P16 was observed. This reduction was consistent across concentrations of 50, 100, and 200 μg/mL for both EV-EC and PT.

The PT/EV-EC treatment exhibited the most significant decrease in P21 and P16 compared to the EV-EC/PT or EV-EC + PT treatments. We did not explore the exact mechanism underlying this observation, as our primary focus was to assess the skin rejuvenation potential of PT and EV-EC, as well as the potential enhancement of this effect through their co-treatment. Future studies should investigate the specific mechanisms influencing the order of co-treatment. Given the maximal effect of PT pre-treatment on reducing cellular senescence in keratinocytes, subsequent analyses should focus on comparing the impact of single treatments of PT or EV-EC with PT/EV-EC treatment.

Transitioning to in vivo studies involving young and aged mice, PT/EV-EC treatment demonstrated the most significant upregulation of HSP70 expression compared to the individual treatments. This upregulation was significantly enhanced with PT/EV-EC in SnCs keratinocytes compared to treatments with PT or EV-EC alone. Additionally, the expression of HSP70 and TNF-α was increased by AA; however, these effects were more prominent with PT/EV-EC treatment. AA increases HSP70 and TNF-α levels [71,72], as well as improving skin health [38]. Thus, our results suggest that PT/EV-EC treatment similarly increases HSP70 expression and decreases TNF-α levels, similar to the effects of well-known antioxidants such as AA.

The expression levels of TNF-α, pSAPK/JNK/SAPK/JNK, pp38/p38, AP-1, and NF-κB were significantly decreased with PT and EV-EC co-treatment in SnCs keratinocytes compared to the individual treatments. In our study, SnCs keratinocytes exhibited a notable reduction in MMP1, MMP3, and MMP9, which are typically elevated by TNF-α, AP-1, and NF-κB during skin aging [19,44]. This decrease was most pronounced with PT/EV-EC treatment.

Treating SnCs keratinocytes with an HSP70 inducer led to reductions in TNF-α, pSAPK/JNK/SAPK/JNK, pp38/p38, and NF-κB levels. Similarly, these factors were decreased by PT/EV-EC treatment, with a more prominent reduction observed when an HSP70 inducer was added. These results suggest the involvement of HSP70 in decreasing TNF-α, SAPK/JNK, p38, and NF-κB with PT/EV-EC treatment. Moreover, the treatment of SnCs keratinocytes with PT/EV-EC led to a decrease in SAPK/JNK and p38 expression comparable to that achieved by treatment with SAPK/JNK or p38 inhibitors. Similarly, AP-1 levels were reduced by PT/EV-EC to a similar extent to that observed with the inhibitors of SAPK/JNK or p38. These findings suggest that PT/EV-EC decreases AP-1 expression via the inhibition of SAPK/JNK and p38.

Heightened oxidative stress, implicated in the augmentation of MMPs or the reduction in collagen synthesis during aging [44], was observed. PT/EV-EC treatment decreased the expression of NOX1, NOX2, and NOX4 to a similar level to the NF-κB inhibitor. These results suggest that PT/EV-EC reduces oxidative stress by inhibiting NF-κB.

Aged keratinocytes induce more inflammation and ECM destruction after UV exposure compared to young keratinocytes [55]. Aging also impacts the interaction between keratinocytes and fibroblasts, exacerbating skin aging pathways by promoting collagen destruction and decreasing collagen synthesis [52,53,54]. This suggests that keratinocytes may contribute to skin aging independently and by influencing fibroblasts.

Administering PT and EV-EC to mice via an MTS in this study led us to infer that PT or EV-EC predominately influences epidermal keratinocytes. In an in vitro model, we utilized CM from keratinocytes to explore how keratinocytes might impact fibroblast behavior. SnCs fibroblasts administered in CM from SnCs keratinocytes exhibited decreased TGF-β1, TGF-β2, and TGF-β3 expression. Conversely, SnCs fibroblasts administered in CM from SnCs keratinocytes treated with PT, EV-EC, and PT/EV-EC demonstrated increased TGF-β1, TGF-β2, and TGF-β3 expression. When CM from NF-κB inhibitor-treated keratinocytes was administered to SnCs fibroblasts, the expression of TGF-β1, TGF-β2, and TGF-β3 increased to levels similar to those observed after treating SnCs fibroblasts with CM from PT/EV-EC-treated SnCs keratinocytes. This suggests that PT/EV-EC increased TGF-β levels by inhibiting NF-κB.

TGF-β, part of the cytokine superfamily, is integral to cell proliferation, differentiation, migration, and ECM production [73], with three isoforms identified in mammals [73]. These isoforms play a crucial role in wound healing by stimulating fibroblasts or immune cells to synthesize collagen [74]. In adult human skin, approximately 80% of the total collagen fibers consist of type I collagen [75], while type III collagen comprises approximately 10% [76]. Both type I and III collagens are key contributors to the ECM, primarily responsible for maintaining the skin’s tensile strength [75].

When SnCs fibroblasts were exposed to CM from SnCs keratinocytes, a reduction in collagen I and III expression was observed. Conversely, SnCs fibroblasts administered CM from SnCs keratinocytes treated with PT, EV-EC, or PT/EV-ECT demonstrated increased collagen I and III expression.

Consistent findings in our in vitro investigation highlighted a significant increase in collagen expression, underscoring the effectiveness of PT/EV-EC co-treatment in enhancing collagen I and III levels. This increase in collagen expression was accompanied by elevated HSP70 levels and reduced expression of TNF-α, pSAPK/JNK/SAPK/JNK, pp38/p38, AP-1, NF-κB, MMP1, MMP3, and MMP9. Additionally, PT/EV-EC treatment led to a decrease in oxidative stress.

PT/EV-EC treatment resulted in the most significant increase in collagen fibers in aged skin compared to the EV-EC/PT or EV-EC + PT treatments. Given the pronounced effects of PT/EV-EC treatment observed in the in vitro model, direct comparisons between the rejuvenation effects of individual and PT/EV-EC treatments were not conducted in the mouse experiments. In the mouse studies, PT/EV-EC treatment resulted in the upregulation of HSP70 expression and downregulation of TNF-α, pSAPK/JNK/SAPK/JNK, pp38/p38, AP-1, and NF-κB expression in aged skin. Moreover, PT/EV-EC treatment mitigated oxidative stress, as evidenced by reduced 8-OHdG, NOX1, NOX2, and NOX4 expression, while concurrently increasing the expression of TGF-β1, TGF-β2, TGF-β3, collagen I, and collagen III in the skin.

PT/EV-EC treatment also reduced MMP1, MMP3, and MMP9 expression levels in aged skin. MMPs are crucial endopeptidases responsible for degrading dermal collagen fibers and BM components [77,78]. Additionally, aging is associated with a decrease in proteins composing the BM, including collagen VII and XVII, nidogen, integrins, and laminin 332, leading to flattening of the papillary pattern of the dermal–epidermal junction [25,79,80,81]. Our study revealed a decrease in the expression of laminin and nidogen in aged skin, but their expression was increased by the administration of PT/EV-EC.

As keratinocytes are anchored to the BM by hemidesmosomes, the integrity of the dermis relies on hemidesmosomes within the BM [82]. PT/EV-EC treatment increased the number of hemidesmosomes in aged skin and mitigated lamina densa disruption. Collagen fiber density can be increased by enhancing collagen synthesis and reducing collagen fiber destruction. PT/EV-EC treatment not only increased TGF-β1, TGF-β2, and TGF-β3, but also decreased MMP1, MMP3, and MMP9. This led to an increase in collagen density in aged skin. Furthermore, both newly synthesized and mature collagen fiber densities were augmented by PT/EV-EC treatment. Since an improvement in both collagen fiber density and BM disruption can enhance skin elasticity, we evaluated skin elasticity, which was also enhanced by PT/EV-EC treatment.

Plant-derived EVs offer distinct advantages over mammalian cell-derived EVs, presenting a more feasible option for large-scale production with rapid manufacturing capabilities [83]. Their reputation for superior biocompatibility [83], minimal cytotoxicity, and fewer side effects [15,83] makes plant-derived EVs a compelling choice in skin rejuvenation studies.

PT has demonstrated low toxicity across cell cultures and animals, with no reported side effects, even at high doses, in humans [84,85,86,87,88]. Endorsed by the European Commission on Diet, Nutrition, and Allergy Food Safety Authority (EFSA), phlorotannins, including PT, are deemed safe as new food additives [89], with a recommended maximal daily dose of 263 mg/day for adults [89].

No cytotoxicity was observed, even at the highest concentration of EV-EC (2 mg/mL), and the mitigation of cellular senescence effects was evident at substantially lower concentrations (25 μL/mL). Balancing safety considerations with the pursuit of rejuvenation, PT/EV-EC emerges as a strategic approach to enhancing collagen and elasticity in aging skin. We applied PT/EV-EC using an MTS to increase the delivery amount to the skin. However, the topical application of PT/EV-EC also showed an increase in collagen fiber in aged skin. Since the enhancing effect was significantly higher when PT/EV-EC was applied via an MTS, it appears that an MTS could be a better option for delivering PT/EV-EC.

In this study, we did not evaluate the long-term effects of PT/EV-EC on aged skin. Moreover, we did not assess the skin rejuvenation effect of PT/EV-EC in humans. Thus, future studies in humans are warranted to determine the skin rejuvenation effect of PT/EV-EC.

## 4. Materials and Methods

### 4.1. Preparation of Phlorotannin

PT was prepared according to the method validated in a previous study [90]. *E. cava* specimens, collected near the coast of Jeju, Korea, were sourced from Aqua Green Technology Co., Ltd. The specimens were thoroughly washed with water and air-dried for 48 h at room temperature before extraction.

Extraction was carried out using 50% ethanol at 85 °C for 12 h. The extracted material was finely ground, and the *E. cava* extracts were subjected to filtration, concentration, and sterilization at 85 °C for 40–60 min. Subsequently, the material was spray-dried to obtain PT [91].

### 4.2. Preparation of EV-EC

*E. cava* (12 g) was dissolved in 360 mL of DW with continuous stirring for 24 h at 50 °C. The mixture was then centrifuged at 3000× *g* for 30 min at room temperature to remove large debris or particulate matter. The resulting supernatant (approximately 38.5 mL) was transferred into an Open-Top Thinwall Ultra-Clear Tube (Beckman Coulter, Brea, CA, USA) and purified by centrifugation at 50,000× *g* for 90 min.

The supernatant, enriched with smaller particles including exosomes, was collected and subjected to ultracentrifugation at 100,000× *g* for 120 min at room temperature to precipitate the exosomes. The resulting exosome pellet was washed by resuspending in DW and again ultracentrifuged at 100,000× *g* for 120 min at room temperature.

The obtained pellet comprised *E. cava*-derived exosomes (EV-EC). The final exosome suspension was resuspended in DW and subjected to freeze-drying.

### 4.3. Nanoparticle-Tracking Analysis

For exosome sample preparation, freeze-dried exosomes were dissolved in 1.5 mL of solvent using a Voltex mixer (WiseMix, Wised, Tägerhardstrasse, Wettingen, Germany) until thoroughly dissolved. The dissolved sample was transferred to a 15 mL conical tube, and an additional 0.5 mL of solvent was added to ensure proper mixing and prevent clumping during dissolution. Further dilutions were prepared using the same solvent.

For NTA analysis, a syringe was loaded with exosome stock and serially diluted solutions (20×, 200×, and 2000×) totaling 1.2 mL. Bubbles were removed from the syringe. The loaded syringe was inserted into the Luer port at the end of the equipment’s suction tube, ensuring contact with the solvent. Once the solvent appeared in the waste tube, the solution was slowly introduced into the chamber by depressing the syringe plunger. The equipment’s laser module was attached, and the analysis was conducted using a NanoSight NS300 (Malvern Panalytic LTD., Malvern, UK).

### 4.4. Dynamic Light Scattering

A clean sample holder was placed into the instrument. The sample was prepared by dispersing the particles in a solvent. Diluted EV-EC was transferred to a cuvette and measured five times at room temperature. The DLS software was initiated, and room temperature was selected. The resulting graphs represent the range of particle sizes in the sample and their corresponding intensities.

### 4.5. Cell Culture

Primary human epidermal keratinocytes (HEKn; American Type Culture Collection [ATCC], Manassas, VA, USA) were cultured in growth medium (GM) consisting of dermal cell basal medium (ATCC) supplemented with a keratinocyte growth kit (ATCC). Cultures were maintained at 37 °C and 5% CO_2_.

Human dermal fibroblasts (CCD-986Sk; ATCC) were cultured in medium composed of Iscove’s Modified Dulbecco′s Medium (Welgene, Gyeongsan, Korea), fetal bovine serum (Gibco^TM^, Thermo Fisher Scientific, Rockford, IL, USA), and 1% penicillin/streptomycin (Welgene). Cells were also maintained at 37 °C and 5% CO_2_.

### 4.6. Cytotoxicity Assay

HEKn cells were seeded into 96-well plates (1 × 10^4^ cells/well) to assess cytotoxicity induced by EV-EC and PT. Upon reaching 100% confluence, cells were treated with varying concentrations of EV-EC (0–5000 μg/mL) or PT (0–5000 μg/mL) for 24 h. Following treatment, the medium containing EV-EC or PT was removed, and cells were washed with Dulbecco’s phosphate-buffered saline (DPBS; Gibco^TM^, Thermo Fisher Scientific).

Subsequently, 10 µL of CCK-8 reagent (TransGene Biotech Co., LTD, Beijing, China) and 90 µL of basal medium (dermal cell basal medium) were added to each well, followed by a 2 h incubation at 37 °C. Optical density was measured at 450 nm using a microplate reader (Multiskan SkyHigh Photometer; Thermo Fisher Scientific). All assays were performed in triplicate.

### 4.7. In Vitro Experimental Design

In vitro experiments were conducted to evaluate the impact of EV-EC and PT on SnCs keratinocytes and fibroblasts and to determine optimal treatment conditions. Keratinocytes were exposed to 50 μM H_2_O_2_ (Sigma-Aldrich, St. Louis, MO, USA) for 2 h to induce senescence. After washing with DPBS, medium was replaced, and cells were incubated for 72 h as previously described [92].

To determine optimal concentrations, SnCs keratinocytes were treated with 0–200 μg/mL of EV-EC or PT and 0–200 μM of AA (A4544; Sigma-Aldrich) for 48 h, and subsequent cell lysates underwent RNA analysis (Figure S2a).

To determine the optimal treatment order, Non-SnCs and SnCs keratinocytes underwent the following treatments (Figure S3a):

(1) Non-SnCs (PBS): PBS treatment for 24 h, subsequent PBS treatment for 24 h.
(2) SnCs (PBS): PBS treatment for 24 h, subsequent PBS treatment for 24 h.
(3) SnCs (EV-EC + PT): Simultaneous treatment with 50 μg/mL EV-EC and 50 μg/mL PT for 24 h, subsequent PBS treatment for 24 h.
(4) SnCs (EV-EC/PT): 50 μg/mL EV-EC treatment for 24 h, subsequent 50 μg/mL PT treatment for 24 h.
(5) SnCs (PT/EV-EC): 50 μg/mL PT treatment for 24 h, subsequent 50 μg/mL EV-EC treatment for 24 h.

Using the optimal concentrations and time points of EV-EC, PT, and AA, the following treatments were assessed (Figure S4):

(1) Non-SnCs (PBS): PBS treatment for 24 h, subsequent PBS treatment for 24 h.
(2) SnCs (PBS): PBS treatment for 24 h, subsequent PBS treatment for 24 h.
(3) SnCs (EV-EC): 50 μg/mL EV-EC treatment for 24 h, subsequent PBS treatment for 24 h.
(4) SnCs (PT): 50 μg/mL PT treatment for 24 h, subsequent PBS treatment for 24 h.
(5) SnCs (PT/EV-EC): 50 μg/mL PT treatment for 24 h, subsequent 50 μg/mL EV-EC treatment for 24 h.
(6) SnCs (AA): 50 μM AA treatment for 24 h, subsequent PBS treatment for 24 h.

After confirming efficacy, evaluations were performed in the following groups: Non-SnCs (PBS), SnCs (PBS), SnCs (EV-EC), SnCs (PT), and SnCs (PT/EV-EC) (Figure S6).

To determine whether increased HSP70 expression inhibited SAPK/JNK, p38, and NF-κB expression, SnCs keratinocytes were treated with GGA, SP600125, SB 203580, or SN50 (Figure S8 and Table S2). Cell lysates and supernatants were collected for protein expression analyses. Supernatants were centrifuged at 3000× *g* for 5 min to remove cells.

Fibroblasts (CCD986-Sk) were exposed to 350 μM H_2_O_2_ for 1.5 h to induce senescence. After washing with DPBS, cells were incubated in fresh medium for 72 h [37]. SnCs fibroblasts were treated with CM for 48 h. CM was prepared by mixing the supernatant from SnCs with fibroblast medium at a ratio of 1:1 (Figures S12 and S15). Cell lysates were collected for subsequent protein expression analyses.

### 4.8. Mouse Model and Maintenance

Female C57BL/6 mice were obtained from Orient Bio (Seongnam, Republic of Korea) in two age groups: 8 weeks and 13 months, 3 weeks old. After a 1-week acclimatization period, the experiments commenced, resulting in two groups aged 9 weeks (young) and 14 months (aged).

Mice were housed in a controlled environment (22 ± 5 °C, 50 ± 10% humidity, and a 12 h light/dark cycle) with unrestricted access to a standard laboratory diet and water.

All experimental protocols were approved by the Center of Animal Care and Use Animal Center Ethics Board (IACUC; approval number: LCDI-2022-0111) of Gachon University and adhered to the guidelines of the Association for Assessment and Accreditation of Laboratory Animal Care.

### 4.9. In Vivo Mouse Experimental Design

In vivo experiments were conducted to assess the efficacy of EV-EC and PT on the skin. To determine optimal concentrations, young and aged mice were randomly divided into six groups (*n* = 3 per group, Figure S16a). Mice received the following treatments on a designated back skin area measuring 2 cm × 2 cm via an MTS:

(1) Young (DW): 200 µL of DW.
(2) Aging (DW): 200 µL of DW.
(3) Aging (EV-EC 0.5 mg/mL): 0.5 mg/mL (200 µL) of EV-EC.
(4) Aging (EV-EC 1 mg/mL): 1 mg/mL (200 µL) of EV-EC.
(5) Aging (PT 0.5 mg/mL): 0.5 mg/mL (200 µL) of PT.
(6) Aging (PT 1 mg/mL): 1 mg/mL (200 µL) of PT.

To determine the optimal order of EV-EC and PT treatment, young and aged mice were randomly divided into five groups (n = 3 per group, Figure S17a). Mice received the following treatments as described above:

(1) Young (DW): Injection of 200 µL of DW, followed by an additional 200 µL of DW after 7 days.
(2) Aging (DW): Injection of 200 µL of DW, followed by an additional 200 µL of DW after 7 days.
(3) Aging (EV-EC + PT): Injection of 1 mg/mL (200 µL) of both EV-EC and PT simultaneously, followed by an additional 200 µL of DW after 7 days.
(4) Aging (EV-EC/PT): Injection of 1 mg/mL (200 µL) of EV-EC, followed by an additional 1 mg/mL (200 µL) of PT after 7 days.
(5) Aging (PT/EV-EC): Injection of 1 mg/mL (200 µL) of PT, followed by an additional 1 mg/mL (200 µL) of EV-EC after 7 days.

To determine the optimal application method of topical and MTS treatments, young and aged mice were randomly divided into four groups (n = 3 per group, Figure S18a). The mice received the following treatments as described above:

(1) Young (DW): Injection of 200 µL of DW, followed by an additional 200 µL of DW after 7 days.
(2) Aging (DW): Injection of 200 µL of DW, followed by an additional 200 µL of DW after 7 days.
(3) Aging (Topical): Topical application of 1 mg/mL (200 µL) of PT, followed by an additional 1 mg/mL (200 µL) of EV-EC after 7 days.
(4) Aging (MTS): Injection of 1 mg/mL (200 µL) of PT, followed by an additional 1 mg/mL (200 µL) of EV-EC after 7 days.

After confirming the efficacy of the treatment method, the effects related to skin rejuvenation were assessed in the following groups: Young (DW), Aged (DW), and Aged (PT/EV-EC) (Figure S19).

Four weeks after the initial treatment, all mice were anesthetized by inhaling 3% isoflurane (HANA Pharm Co., Ltd., Seoul, Republic of Korea) in the presence of 1.5% O_2_. The treated skin regions were then shaved, and skin tissues were collected for analysis.

To determine whether the skin elasticity of aged skin was altered by PT/EV-EC treatment, skin elasticity measurements were taken both before treatment and before skin tissue collection. Skin elasticity was measured using skin measurement diagnostics (API-100; Aram Huvis Co., Ltd., Seongnam, Republic of Korea), averaging five measurements per mouse.

### 4.10. TEM Analysis

#### 4.10.1. EV-EC Sample Preparation

A drop of the sample was placed on a Formvar-carbon-coated grid for 15 s. The excess droplet was removed using filter paper, and a drop of 1% uranyl acetate was applied for 15 s. After removing the excess uranyl acetate using filter paper, the grid was washed with a drop of DW [93,94].

#### 4.10.2. Skin Sample Preparation

Skin specimens were fixed for 12 h in a solution containing 2% glutaraldehyde and 2% paraformaldehyde in 0.1 M phosphate buffer (pH 7.4). Following fixation, specimens were washed in 0.1 M phosphate buffer and post-fixed with 1% OsO_4_ in 0.1 M phosphate buffer for 2 h. Samples were dehydrated using an ascending ethanol series (50, 60, 70, 80, 90, 95, and 100%) for 10 min each and infiltrated with propylene oxide for 10 min.

Fixed specimens were embedded using a Poly/Bed 812 kit (Polysciences, Warrington, PA, USA) and polymerized in an electron microscope oven (TD-700, DOSAKA, Kyoto, Japan) at 65 °C for 12 h. Using an ultramicrotome equipped with a diamond knife, blocks were sectioned into 200 nm semi-thin sections and stained with toluidine blue for optical microscopy. Regions of interest were then cut into 80 nm thin sections using the ultramicrotome, placed on copper grids, and double-stained with 3% uranyl acetate for 30 min followed by 3% lead citrate for 7 min.

#### 4.10.3. TEM Imaging

Images were captured using a transmission electron microscope (JEM-1011, JEOL, Tokyo, Japan) operating at an acceleration voltage of 80 kV and equipped with a Mega-view III CCD camera (Soft imaging system, Münster, Germany). The size of the EV-EC was measured using TEM images within ImageJ software, Version 1.54i (National Institutes of Health, NIH, Maryland, MD, USA).

### 4.11. RNA Extraction, cDNA Synthesis, and Quantitative Real-Time Polymerase Chain Reaction (qRT-PCR)

RNA isolation from cell lysates was performed utilizing RNAiso reagent (TAKARA, Tokyo, Japan) following the manufacturer’s protocol. The resulting RNA pellet was air-dried for 10–15 min at room temperature and reconstituted in diethylpyrocarbonate-treated water (Biosesang, Seongnam, Republic of Korea). Quantification and evaluation of RNA quality were conducted using a NanoDrop spectrophotometer (Thermo Fisher Scientific).

For cDNA synthesis, 1 µg of RNA was combined with oligo dT primers and dNTPs in RNase-free DW (TAKARA) and incubated at 65 °C for 5 min. Reverse transcriptase (TAKARA) and RNase inhibitor (TAKARA) were subsequently added for 45 min at 42 °C, followed by a final denaturation step at 95 °C for 5 min using a thermal cycler (Bio-Rad, Hercules, CA, USA).

For qRT-PCR, a total volume of 10 µL was used, comprising 2.5 µg of cDNA template, 5 µL of ROX Plus SYBR Green Premix (TAKARA), 0.8 µL of each reverse and forward primer (Table S3), and 1.7 µL of DW. qRT-PCR was conducted on a QuantStudio 3 instrument (Thermo Fisher Scientific) with an initial denaturation at 95 °C for 10 min, followed by 40 cycles of 95 °C for 15 s, 60 °C for 1 min, and 95 °C for 15 s. A melting analysis spanning 60–95 °C at a rate of increase of 0.075 °C/second was performed. Gene expression levels were quantified using the comparative CT method (ΔΔCT), with mRNA levels normalized to *Actb* and expressed relative to the Non-SnCs/PBS group.

### 4.12. Protein Isolation

Cells were rinsed with PBS, detached using a cell scraper (SPL, Seoul, Republic of Korea), and suspended in radioimmunoprecipitation assay (RIPA) lysis buffer supplemented with protease and phosphatase inhibitors (EzRIPA Buffer Kit; ATTO Corporation, Tokyo, Japan). Skin tissue samples were homogenized using a bead homogenizer (Allsheng Instrument, Hangzhou, China) at 6.0 m/s for 5 cycles (40 s running, 1 min interruption per cycle) in RIPA buffer. Homogenates were then incubated on ice for 15 min to facilitate cell lysis and protein solubilization.

Following homogenization, both cell and tissue samples were sonicated and centrifuged at 14,000× *g* for 15 min at 4 °C to separate soluble proteins from insoluble debris and organelles. Protein concentrations were determined using a bicinchoninic acid assay kit (Thermo Fisher Scientific).

### 4.13. Western Blot

For Western blot analysis, proteins (50 µg of EV-EC and PT or 30 µg of cell lysates and skin tissues) were denatured in 4× LDS sample buffer (Thermo Fisher Scientific) and a 10× sample reducing agent (Thermo Fisher Scientific) for 10 min at 70 °C. Separation employed 10% or 12% SDS-PAGE with MOPS buffer (Invitrogen, Waltham, MA, USA), followed by transfer to polyvinylidene difluoride membranes (Merck Millipore, Burlington, MA, USA). Membranes were blocked with 5% skim milk solution (LPS solution, Daejeon, Republic of Korea), probed with primary antibodies (Table S4), and incubated with horseradish peroxidase-conjugated secondary antibodies (Vector Laboratories, Burlingame, CA, USA). Protein bands were visualized using enhanced chemiluminescence (Cytiva^TM^, Marlborough, MA, USA) with a ChemiDoc Imaging System (Bio-Rad). Bands were quantified using ImageJ software and normalized to β-actin bands. Expression values represent the experimental samples from aged mice relative to the mean of the first bar in the graphs [95].

### 4.14. Enzyme-Linked Immunosorbent Assay

To quantify MMP1, MMP3, MMP9, 8-OHdG, TGF-β1, TGF-β2, and TGF-β3, 96-well microplates were coated with a 100 nM carbonate and bicarbonate-mixed buffer (pH 9.6) and incubated overnight at 4 °C. Microplates were washed with PBS containing 0.1% Tween 20 (TPBS) and blocked with 5% skim milk. Following another PBS wash, each well received 30 μg of protein samples and was incubated overnight at 4 °C. Wells were probed with primary antibodies (Table S3) and incubated with peroxidase-conjugated secondary antibodies (Vector Laboratories). For detection, tetramethylbenzidine solution was added for 15–20 min at room temperature, and the reaction was stopped with 2 N sulfuric acid. Optical density was measured at a wavelength of 450 nm with a Multiskan SkyHigh microplate reader (Thermo Fisher Scientific).

### 4.15. Sectioning of Paraffin-Embedded Skin Tissue

Skin tissues were fixed in cold 4% paraformaldehyde (Sigma-Aldrich) for 48 h, dehydrated in increasing concentrations of ethanol, cleared with xylene, and embedded in paraffin using a tissue processor (Sakura Seiki Co., Ltd., Tokyo, Japan).

The paraffin-embedded tissue blocks were precision-sectioned into 7 μm thick slices using a microtome (Thermo Fisher Scientific). For adhesion, sections were air-dried and then incubated overnight at 60 °C. Subsequently, sections underwent deparaffinization and rehydration through sequential immersion in xylene (Duksan, Seoul, Republic of Korea) and a 70–100% ethanol gradient (Duksan).

### 4.16. Immunocytochemistry (ICC)

Cultured cells (1 × 10^4^ cells/well) were prepared following the procedure outlined in Section 4.6 and seeded into 8-well Lab-Tek II chamber slides (Nunc™, Sigma-Aldrich). After washing with PBS, cells were fixed in 4% paraformaldehyde (PFA; Sigma-Aldrich) at room temperature for 15 min. Subsequently, cells were washed with PBS and blocked with normal serum solution for 1 h, followed by overnight incubation with primary antibodies (Table S3) at 4 °C. Cells were again washed and incubated with secondary antibodies (Invitrogen) at room temperature for 1 h in the dark. Following a final wash, nuclei were stained with 1 µg/mL of 4′,6-diamidino-2-phenylindole (DAPI; Sigma-Aldrich) for 30 s. Coverslips were mounted using Vectashield mounting solution (Vector Laboratories), and analysis was conducted using a confocal microscope (Carl Zeiss 700; Carl Zeiss, Jena, Germany) at the Core-facility for Cell to In vivo imaging.

Colocalization analysis of AP-1 and NF-κB was performed to assess fluorophore overlap using Zen software (Carl Zeiss). For collagen I and III quantification, fluorescence intensities in the images were also analyzed using Zen software, applying a thresholding algorithm to identify individual regions of interest (green). Manual adjustments ensured that only regions of interest were included in the analysis.

### 4.17. Microscopic Analysis of Tissue Sections

#### 4.17.1. Sample Preparation

Skin tissues were fixed in cold 4% paraformaldehyde (Sigma-Aldrich) for 48 h, dehydrated in increasing concentrations of ethanol, cleared with xylene, and embedded in paraffin using a tissue processor (Sakura Seiki Co., Ltd., Tokyo, Japan). The paraffin-embedded tissue blocks were precision-sectioned into 7 μm thick slices using a microtome (Thermo Fisher Scientific). For adhesion, sections were air-dried and incubated overnight at 60 °C.

Subsequently, sections underwent deparaffinization and rehydration through sequential immersion in xylene (Duksan, Seoul, Republic of Korea) and a 70–100% ethanol gradient (Duksan) for both the sectioning of paraffin-embedded skin tissue and the preparation of slides for immunohistochemistry.

#### 4.17.2. Immunohistochemistry

For antigen retrieval, slides were boiled in sodium citrate buffer (pH 6.0) using a microwave oven for 5 min, cooled in DW, and washed with PBS. Next, slides were treated with 3% H_2_O_2_ in PBS for 10 min at room temperature, followed by three PBS washes. Slides were then incubated with 0.1% Triton X-100 in PBS for 5 min at room temperature washed with PBS, and incubated with 0.01% normal serum solution for 1 h at room temperature to block nonspecific binding. Blocked slides were further incubated overnight at 4 °C with primary antibodies (Table S3) and for an additional 1 h at room temperature. Washed slides were incubated with biotinylated secondary antibodies (Vector Laboratories) for 1 h at room temperature, washed with PBS, and incubated with ABC reagent (Vector Laboratories) according to the manufacturer’s instructions. After washing, slides were developed with 3,3′-diaminobenzidine solution (Sigma-Aldrich) for 15 min to obtain a brown color. For counter-staining, slides were incubated with hematoxylin (KPNT, Cheongju, Republic of Korea) for 30 s, washed with DW, and mounted using DPX mounting solution (Sigma-Aldrich). Specimens were imaged using a slide scanner (Motic Scan Infinity 100; Motic, Beijing, China), and ImageJ software was used to measure the intensity and count positive signals [96].

#### 4.17.3. Masson Trichrome Staining

To visualize collagen fibers in skin tissues, the previously fixed and paraffinized samples were immersed in Bouin solution (Scytek Laboratories, West Logan, UT, USA) and heated for 1 h at 60 °C, followed by washing with DW. The sections underwent sequential treatment: iron hematoxylin (KPNT) for 10 min, Biebrich scarlet acid fuchsin solution (Scytek Laboratories, West Logan, UT, USA) for 2 min, phosphomolybdic-phosphotungstic acid solution (Scytek Laboratories) for 15 min, and aniline blue solution (Scytek Laboratories) for 3 min, all at room temperature. Subsequently, the stained slides were sealed with DPX mounting solution and examined using an optical microscope (Olympus, Tokyo, Japan) equipped with a slide scanner (Motic). Collagen fiber density analysis was performed on all images using ImageJ software [97].

#### 4.17.4. Herovici’s Staining

In skin tissue samples, we differentiated between mature collagen fibers and newly synthesized collagen fibers using a Herovici Collagen Stain Kit (Scytek Laboratories). Deparaffinized slides were incubated in Weigert’s iron hematoxylin for 8 min to highlight the nuclei, followed by washing with tap water and DW. Slides were then exposed to the Herovici solution for 2 min and sealed with DPX mounting solution. For microscopic observation and imaging, a slide scanner (Motic) was utilized. Newly synthesized collagen fibers stained blue, while mature collagen fibers appeared red [41,42]. Quantitative staining analysis was conducted using ImageJ software.

### 4.18. Statistical Analysis

Statistical significance was assessed using the Kruskal–Wallis test, with group comparisons conducted via the Mann–Whitney U test. Results are presented as mean ± standard deviation. Statistical analyses were performed using SPSS version 22 (IBM Corporation, Armonk, NY, USA). Significance levels are specified in each figure legend.

## 5. Conclusions

In summary, our investigation into the efficacy of PT and EV-EC treatments yielded promising outcomes for addressing various aspects of skin aging. By targeting intrinsic and extrinsic factors influencing collagen deposition and elasticity, our data highlighted the complex interaction between keratinocytes and fibroblasts, particularly under conditions of senescence and UV exposure.

**Figure 8:**
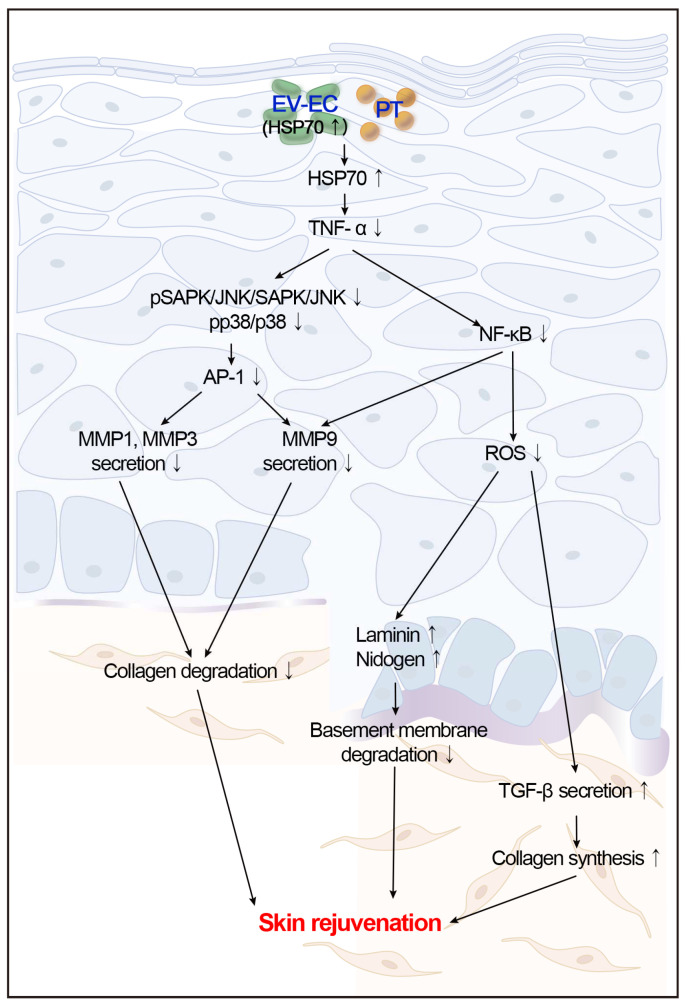
Summary of rejuvenation effects of sequential PT and EV-EC treatments on aged skin. Upon treatment, HSP70 expression increased, while expression of TNF-α, pSAPK/JNK, pp38, AP-1, and NF-κB decreased, concomitant with reduced oxidative stress. Similarly, secretion of MMP1, MMP3, and MMP9 declined. Subsequently, expression of TGF-β1, TGF-β2, TGF-β3, collagen I, collagen III, and basement membrane proteins (laminin and nidogen) was elevated. These changes facilitated accumulation of collagen fibers and improvement in elasticity in PT/EV-EC-treated skin of aged mice.

The synergistic effects of PT and EV-EC, validated through comprehensive in vitro and in vivo studies, resulted in a significant reduction in specific senescence markers, pro-inflammatory factors, and MMP expression. Moreover, we observed a notable increase in collagen density and improvements in elasticity. Notably, the observed modulation of HSP70 and TGF-β pathways suggests the combined inhibition of oxidative stress and inflammation, as illustrated in Figure 8. These findings underscore the potential of PT/EV-EC treatment as a strategic and safe approach for enhancing collagen production and restoring elasticity in aging skin.

While further exploration is warranted to elucidate the precise mechanisms underlying these effects, our study provides compelling evidence supporting the clinical translation of PT/EV-EC treatment in addressing skin aging. This research opens new avenues for future investigations aimed at developing a deeper mechanistic understanding and the development of innovative skin rejuvenation therapies.

## Data Availability

All data are contained within the article.

## Supplementary Materials

The following supporting information can be downloaded at: https://www.mdpi.com/article/10.3390/md22050223/s1. Figure S1. Assessment of in vitro cytotoxicity induced by EV-EC and PT treatments. Figure S2. Optimization of EV-EC, PT, and AA concentrations for SnCs keratinocytes. Figure S3. Determination of optimal EV-EC and PT treatment order in SnCs keratinocytes. Figure S4. Schematic representation of the in vitro model for keratinocytes utilizing optimal concentrations and time points of EV-EC, PT, or AA. Figure S5. Modulation of HSP70 and TNF-α by EV-EC, PT, PT/EV-EC, and AA in H_2_O_2_-induced SnCs keratinocytes. Figure S6. Overexpression of HSP70 by PT/EV-EC in H_2_O_2_-induced SnCs keratinocytes. Figure S7. Inhibition of SAPK/JNK and p38 by PT/EV-EC in H_2_O_2_-induced SnCs keratinocytes. Figure S8. Induction of inhibition of SAPK/JNK and p38 in SnCs keratinocytes treated with EV-EC and PT. Figure S9. Regulation of NOX1, NOX2, and NOX4 in SnCs keratinocytes treated with EV-EC and PT. Figure S10. Schematic diagram of the in vitro model for fibroblasts exposed to CM from SnCs keratinocytes treated with EV-EC, PT, or PT/EV-EC. Figure S11. Upregulation of collagen I and III in SnCs fibroblasts exposed to EV-EC- and PT-treated SnCs keratinocyte CM. Figure S12. Schematic diagram of the in vitro model for keratinocytes and fibroblasts exposed to CM from SnCs keratinocytes treated with PT/EV-EC or following NF-κB inhibition. Figure S13. Upregulation of ROS and TGF-β levels in SnCs keratinocytes and SnCs fibroblasts exposed to CM from SnCs keratinocytes treated with PT/EV-EC or following NF-κB inhibition. Figure S14. Determination of the optimal treatment dosage of PT and EV-EC in the skin of aged mice. Figure S15. Determination of the optimal EV-EC and PT treatment order in the skin of aged mice. Figure S16. Determination of the optimal application method of topical or MTS treatment in the skin of aged mice. Figure S17. Mouse model for efficacy verification of EV-EC and PT treatment in skin rejuvenation. Figure S18. Regulation of HSP70, TNF-α, MAPK, AP-1, and NF-κB expression in the skin of the aged mice treated with PT/EV-EC. Figure S19. Regulation of NOX1, NOX2, NOX4, collagen I, and collagen III levels in the skin of aged mice treated with PT/EV-EC. Table S1. Dynamic light scattering analysis and nanoparticle-tracking analysis of the EV-EC utilized in this study. Table S2. List of overexpression and inhibitors. Table S3. Primers utilized for quantitative reverse transcription–polymerase chain reaction. Table S4. Antibodies utilized for Western blot, ELISA, ICC, and IHC.
