# Supplementary material for: Extracellular Vesicles from Ecklonia cava and Phlorotannin Promote Rejuvenation in Aged Skin
Source: Mar Drugs. 2024 May 15;22(5):223. doi: 10.3390/md22050223 (PMC11123375; doi:10.3390/md22050223)
Supplement: Supplementary file 1 [file marinedrugs-22-00223-s001.zip › marinedrugs-2879706-supplementary.pdf]

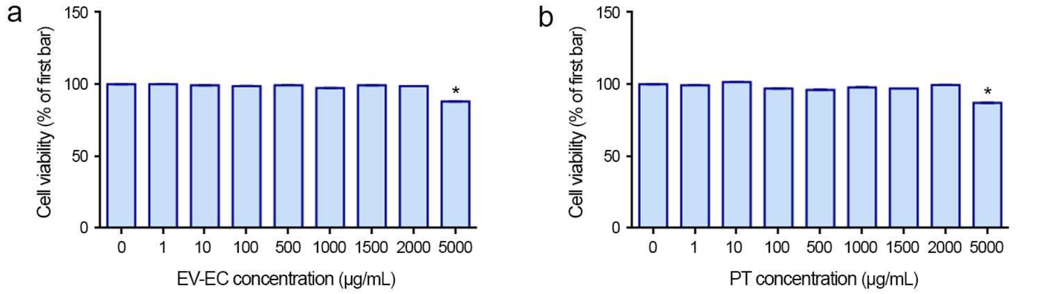

Figure S1. Assessment of *in vitro* cytotoxicity induced by EV-EC and PT treatments. (**a, b**) Cytotoxic effects were examined across various concentrations (1–5,000 μg/mL) of EV-EC and PT on keratinocytes following a 24 h incubation. No cytotoxicity was observed even at the highest concentration of 2,000 μg/mL for both EV-EC and PT. However, cytotoxic effects were noted at 5,000 μg/mL. Data are presented as mean ± SD of three independent experiments. *, $p < 0.05$, vs. first bar; EV-EC, extracellular vesicles from *Ecklonia cava*; PT, phlorotannin; SD, standard deviation.

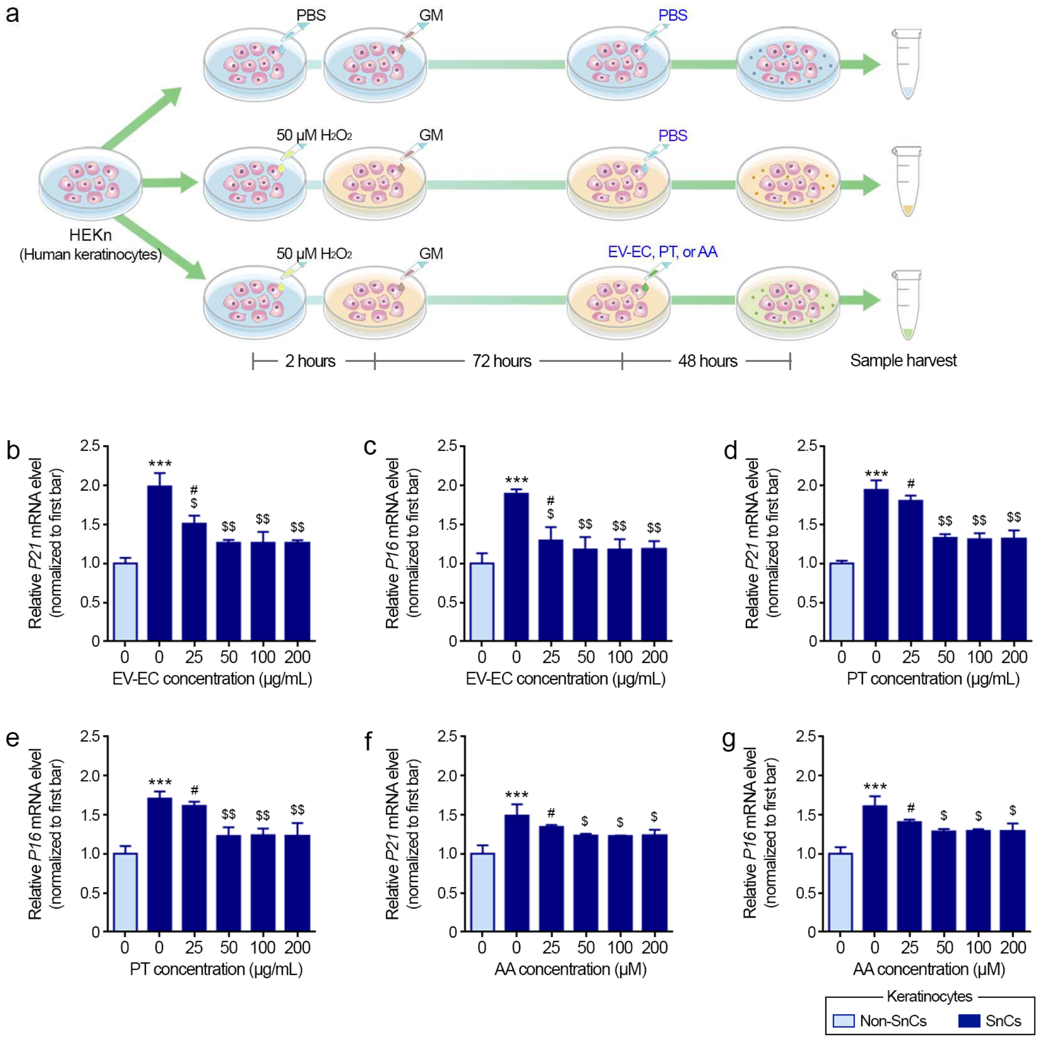

Figure S2. Optimization of EV-EC, PT, and AA concentrations for SnCs keratinocytes. (**a**) Experimental design schematic illustrating the induction of SnCs in keratinocytes followed by treatment with EV-EC, PT, and AA. Keratinocytes underwent a 2 h treatment with 50 μM H_2O_2, followed by a 72 h treatment with GM to induce SnCs. Subsequently, cells were treated with 25–200 μg/mL of EV-EC or PT, or 25–200 μM AA for 48 h. (**b, c**) qRT-PCR assessment of senescence markers *P21* and *P16* in EV-EC-treated SnCs keratinocytes. (**d, e**) qRT-PCR assessment of *P21* and *P16* in PT-treated SnCs keratinocytes. (**f, g**) qRT-PCR assessment of P21 and P16 in AA-treated SnCs

keratinocytes. Data were normalized to ACTB, and expression levels were determined using the comparative cycle threshold method relative to the first bar in each graph. Data are presented as mean ± SD of three independent experiments. ***, $p < 0.001$, first bar vs. second bar; $, $p < 0.05$, second bar vs. third, fourth, fifth, and sixth bars; $$, $p < 0.01$, second bar vs. fourth, fifth, and sixth bars; #, $p < 0.05$, third bar vs. fourth, fifth, and sixth bars (Mann–Whitney U test). AA, ascorbic acid; GM, growth medium; HEKn, human epidermal keratinocyte; Non-SnCs, non-senescent cells; PBS, phosphate-buffered saline; qRT-PCR, quantitative real-time polymerase chain reaction; SnCs, senescent cells.

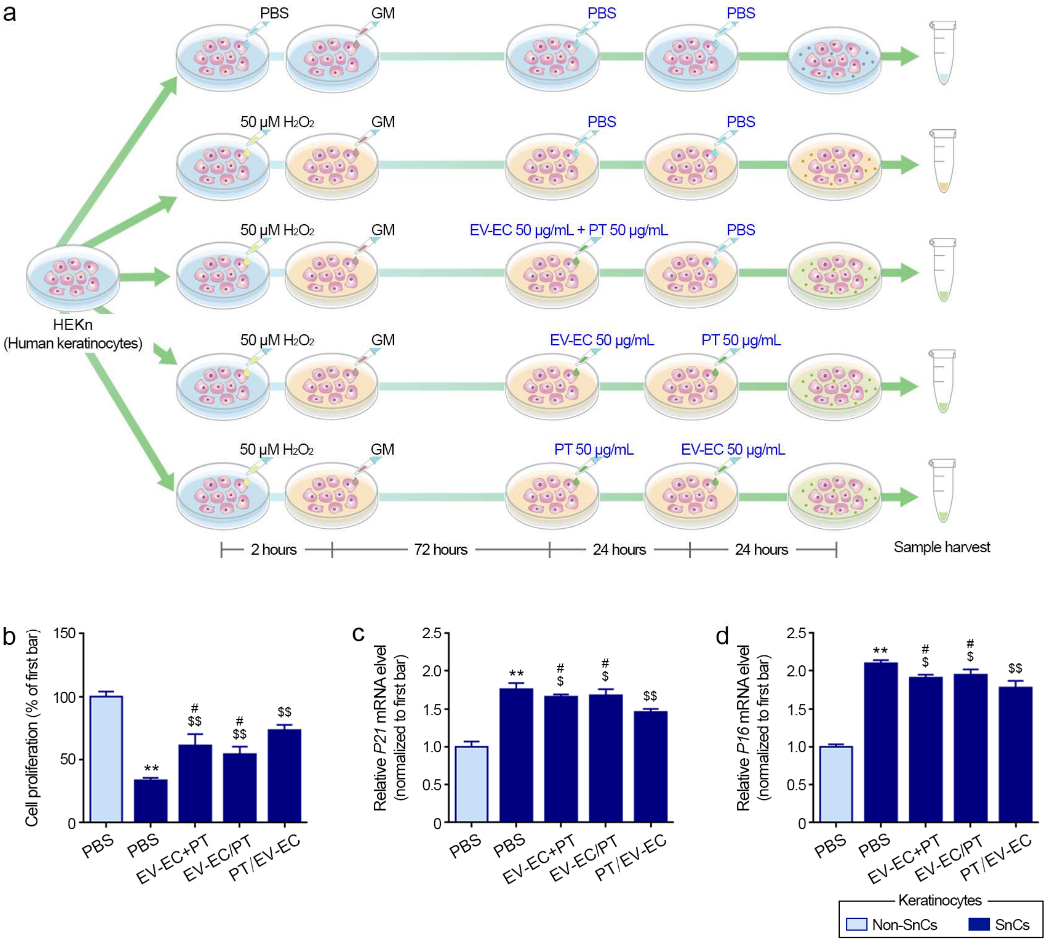

Figure S3. Determination of optimal EV-EC and PT treatment order in SnCs keratinocytes. (**a**) Experimental design schematic illustrating the treatment conditions. Keratinocytes were exposed to PBS as a control (Non-SnCs(PBS) group). SnCs keratinocytes, induced by treatment with 50 μM H_2O_2 followed by GM, were treated with EV-EC and/or PT in the following groups: (1) SnCs(PBS): SnCs keratinocytes treated with PBS. (2) SnCs(EV-EC+PT): SnCs keratinocytes simultaneously treated with EV-EC (50 μg/mL) and PT (50 μg/mL). (3) SnCs(EV-EC/PT): SnCs keratinocytes pre-treated with EV-EC (50 μg/mL) and subsequently treated with PT (50 μg/mL). (4) SnCs(PT/EV-EC): SnCs keratinocytes pre-treated with PT (50 μg/mL) and subsequently treated with EV-EC (50 μg/mL). (**b**) Cell proliferation assessed by CCK8 assay. (**c, d**) qRT-PCR assessment of P21 and P16 (senescence markers). Data are presented as mean ± SD of three independent experiments. **, $p < 0.01$, first bar vs. second bar; $, $p < 0.05$ and $$, $p < 0.01$, second bar vs. third, fourth, and fifth bars; #, $p < 0.05$, fifth bar vs. third or fourth bars (Mann–Whitney U test).

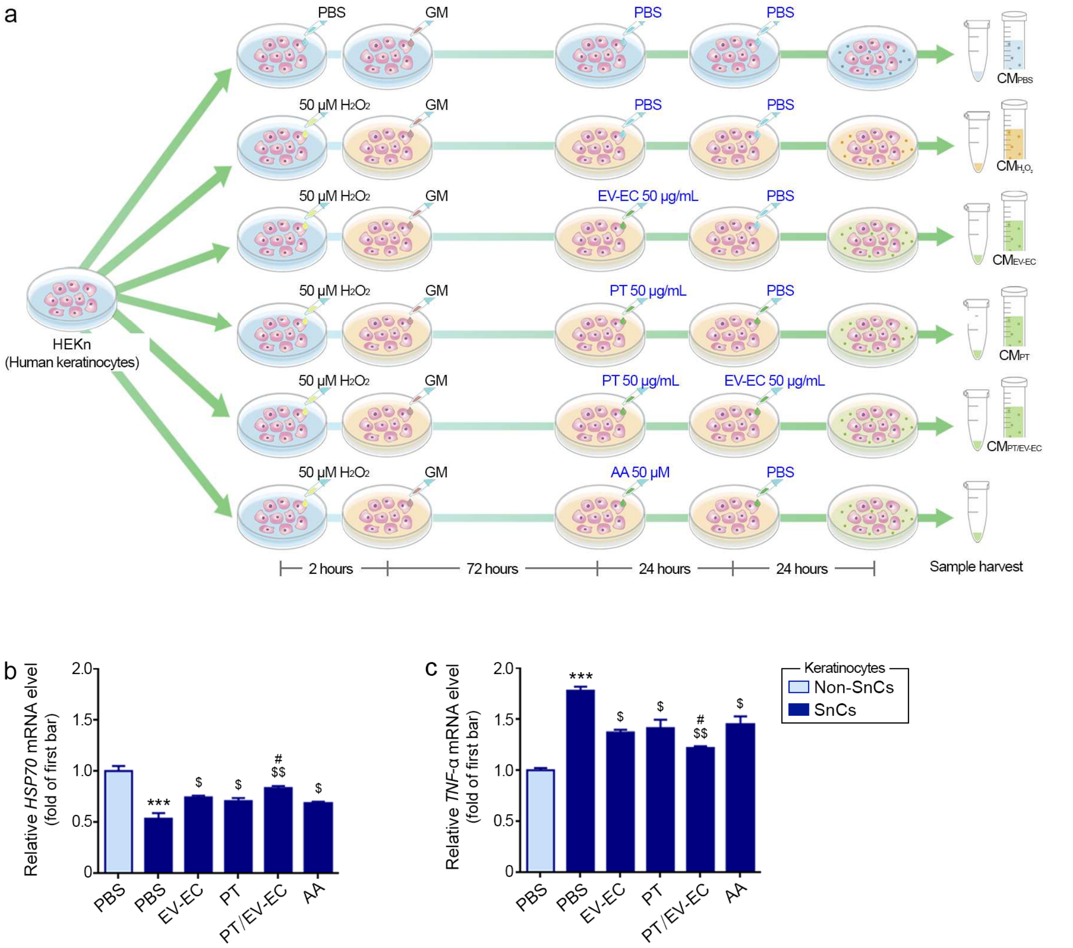

Figure S4. Schematic representation of the *in vitro* model for keratinocytes utilizing optimal concentrations and time points of EV-EC, PT, or AA. (**a**) Keratinocytes were exposed to PBS as a control (Non-SnCs(PBS) group). Cells were treated with 50 μM H_2O_2 for 2 h to induce senescence and GM was replaced for 72 h. SnCs keratinocytes were treated with EV-EC, PT, or AA in the following groups: (1) SnCs(PBS): SnCs keratinocytes treated with PBS. (2) SnCs(EV-EC): SnCs keratinocytes pre-treated with EV-EC (50 μg/mL) for 24 h and then treated with PBS for 24 h. (3) SnCs(PT): SnCs keratinocytes pre-treated with PT (50 μg/mL) for 24 h and subsequently treated with PBS for 24 h. (4) SnCs(PT/EV-EC): SnCs keratinocytes pre-treated with EV-EC (50 μg/mL) for 24 h and then treated with PT (50 μg/mL) for 24 h. (5) SnCs(AA): SnCs keratinocytes pre-treated with AA (50 μM) for 24 h and then treated with PBS for 24 h. Cell lysates were collected for further analysis. (**b, c**) qRT-PCR assessment of HSP70 and TNF-α. Data were normalized to ACTB, and expression levels were determined using the comparative cycle threshold method relative to the first bar in each graph. Data are presented as mean ± SD of three independent experiments. ***, $p < 0.001$, first bar vs. second bar; $, $p < 0.05$, second bar vs. third, fourth, and fifth bars; $$, $p < 0.01$, second bar vs. fifth bar; #, $p < 0.05$, fifth bar vs. third, fourth, and sixth bars (Mann–Whitney U test).

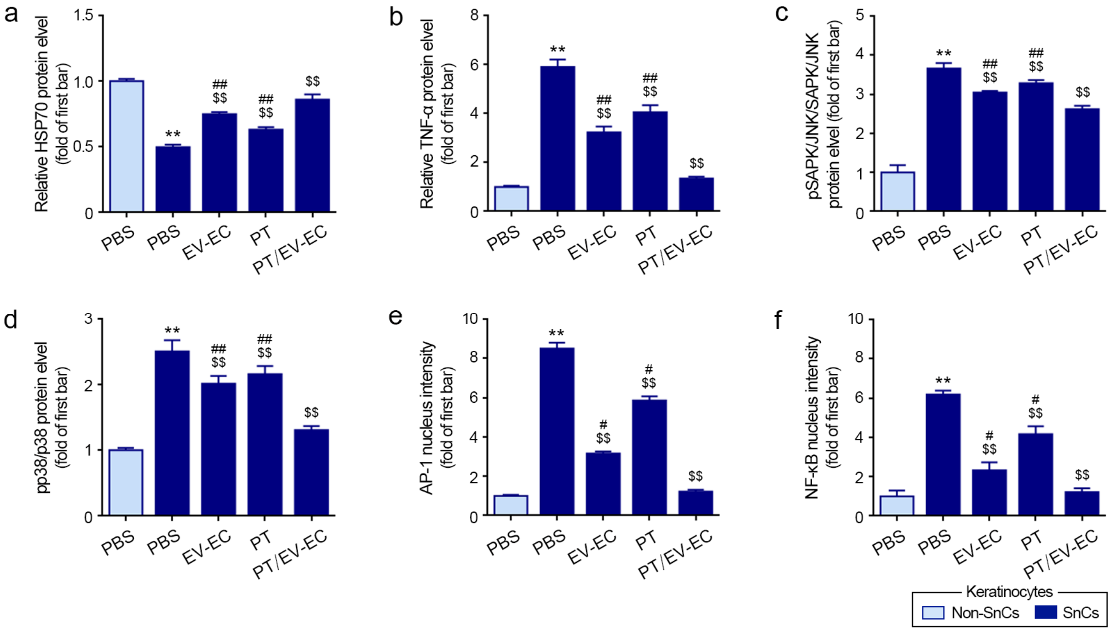

Figure S5. Modulation of HSP70, TNF-α, MAPK, AP-1, and NF-κB by EV-EC and PT in H_2O_2-induced SnCs keratinocytes. (**a, b**) Quantitative assessment of the Western blot data presented in Figure 2a. (**c, d**) Quantitative assessment of the Western blot data presented in Figure 2b. (**e, f**) Quantification of the intensity of the green nuclear signals in Figure 2c. Data are presented as mean ± SD of three independent experiments. **, $p < 0.01$, first bar vs. second bar; $$, $p < 0.01$, second bar vs. third, fourth, and fifth bars; #, $p < 0.05$ and ##, $p < 0.01$, fifth bar vs. third or fourth bars (Mann–Whitney U test). AP-1, activator protein-1; NF-κB, nuclear factor kappa-light-chain-enhancer of activated B cells; pp38, phosphorylated p38; pSAPK/JNK, phosphorylated SAPK/JNK; SAPK/JNK, stress-activated protein kinases/Jun-amino-terminal kinase.

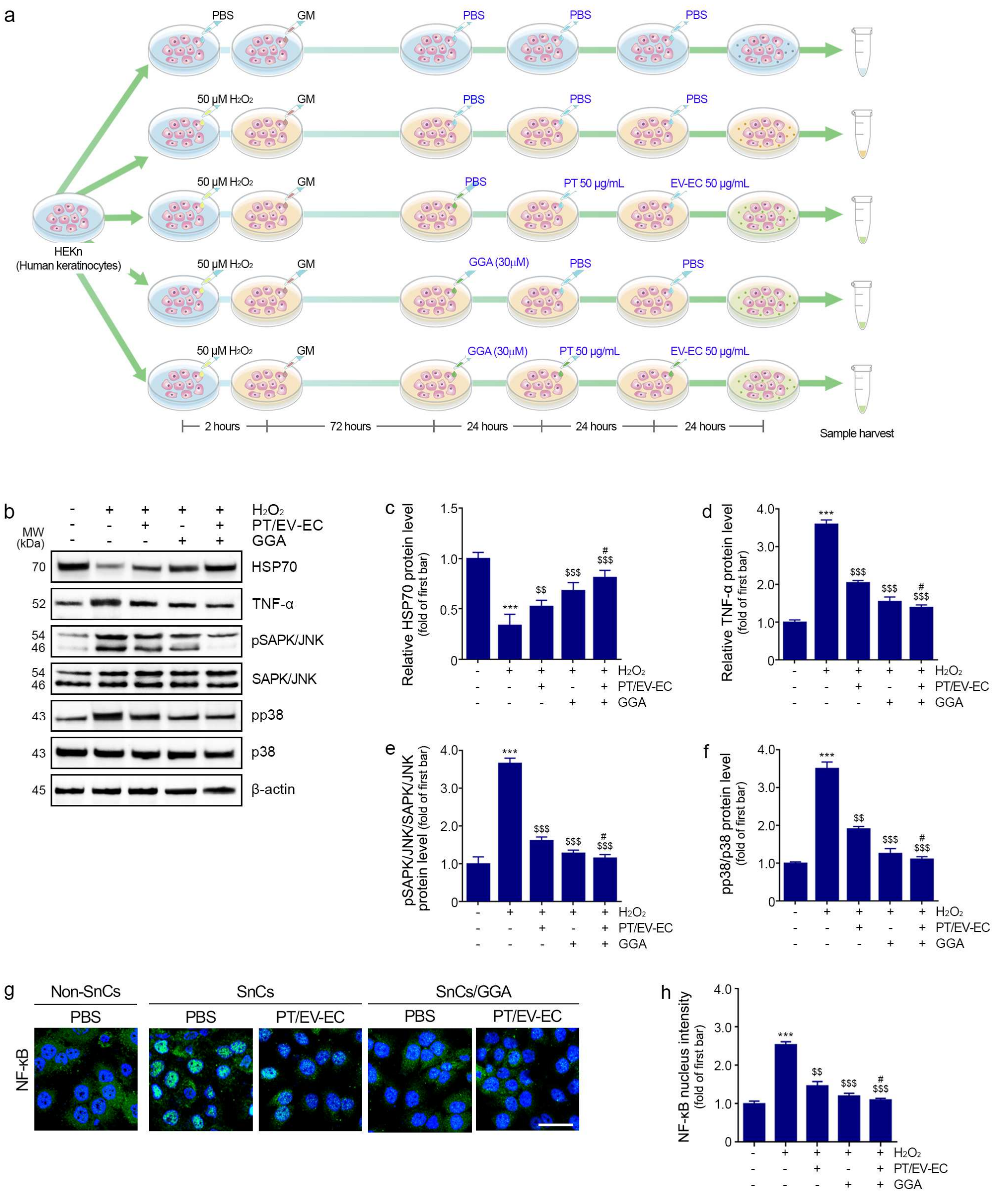

Figure S6. Overexpression of HSP70 by PT/EV-EC in H_2O_2-induced SnCs keratinocytes. (**a**) Keratinocytes were exposed to PBS as a control (Non-SnCs(PBS) group). Cells were treated with 50 μM H_2O_2 for 2 h to induce senescence and GM was replaced for 72 h. SnCs keratinocytes were treated with PT/EV-EC in the following groups: (1) SnCs(PBS): SnCs keratinocytes treated with PBS. (2) SnCs(PT/EV-EC): SnCs keratinocytes treated with PBS for 24 h and pre-treated with PT (50 μg/mL) for 24 h and then treated with EV-EC (50 μg/mL) for 24 h. (3) SnCs (GGA): SnCs keratinocytes treated with GGA (30 μM) for 24 h and pre-treated with PBS for 24 h and then treated with PBS 24 h. (4) SnCs (GGA/PT/EV-EC): SnCs keratinocytes pre-treated with GGA for 24 h and pre-treated with PT for 24 h and then treated with EV-EC 24 h. Cell lysates and supernatants (CM) were collected for further analysis. (**b**) Western blot analysis of HSP70, TNF-α, pSAPK/JNK, total SAPK/JNK, pp38, total p38, and β-actin levels in Non-SnCs and SnCs keratinocytes treated with PBS, PT/EV-EC, GGA, or GGA/PT/EV-EC. (**c-f**) Quantitative assessment of the Western blot data presented in Figure S6b. (**g**) ICC analysis of NF-κB expression (green) in Non-SnCs and SnCs keratinocytes (nuclei: blue; scale bar = 50 μm). (**h**) Quantification of the intensity of the nuclear green signal in Figure s6g. Data are presented as mean ± SD of three independent experiments. ***, $p < 0.001$, first bar vs. second bar; $$, $p < 0.01$ and $$$, $p < 0.001$, second bar vs. third, fourth, and fifth bars; #, $p < 0.05$, fifth bar vs. third or fourth bars (Mann–Whitney U test). AP-1, activator protein-1; NF-κB, nuclear factor kappa-light-chain-enhancer of activated B cells; pp38, phosphorylated p38; pSAPK/JNK, phosphorylated SAPK/JNK; SAPK/JNK, stress-activated protein kinases/Jun-amino-terminal kinase.

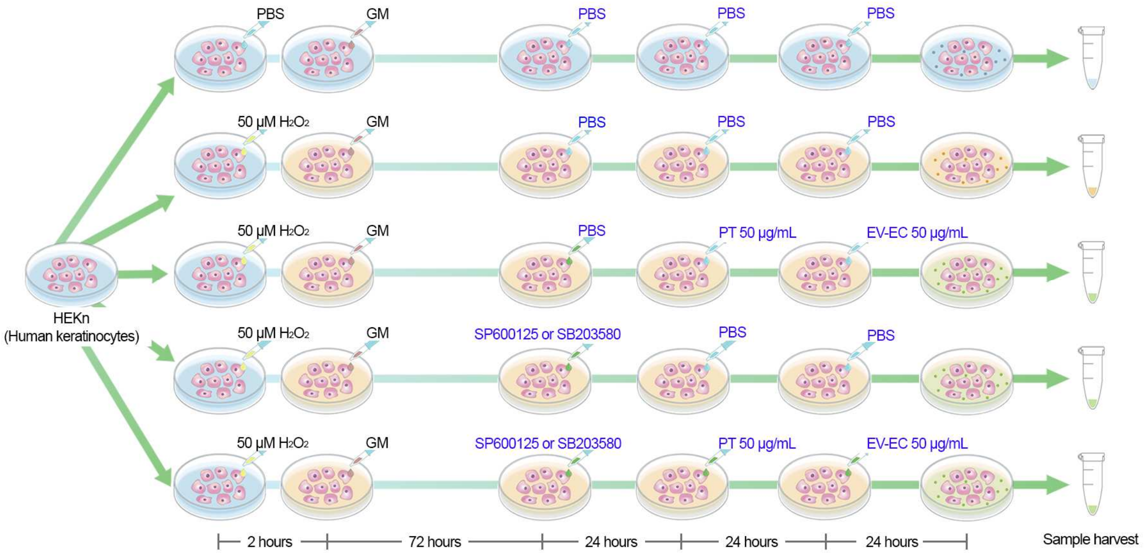

Figure S7. Inhibition of SAPK/JNK and p38 by PT/EV-EC in H_2O_2-induced SnCs keratinocytes. Keratinocytes were exposed to PBS as a control (Non-SnCs(PBS) group). Cells were treated with 50 μM H_2O_2 for 2 h to induce senescence and GM was replaced for 72 h. SnCs keratinocytes were treated with PT/EV-EC in the following groups: (1) SnCs(PBS): SnCs keratinocytes treated with PBS. (2) SnCs(PT/EV-EC): SnCs keratinocytes treated with PBS for 24 h and pre-treated with PT (50 μg/mL) for 24 h and then treated with EV-EC (50 μg/mL) for 24 h. (3) SnCs(SP600125 or SB203580): SnCs keratinocytes treated with SP600125 (50 μM) or SB203580 (20 μM) for 24 h and pre-treated with PBS for 24 h and then treated with PBS 24 h. (4) SnCs(SP600125/PT/EV-EC or SB203580/PT/EV-EC): SnCs keratinocytes pre-treated with SP600125 or SB203580 for 24 h and pre-treated with PT for 24 h and then treated with EV-EC 24 h. Cell lysates and supernatants (CM) were collected for further analysis.

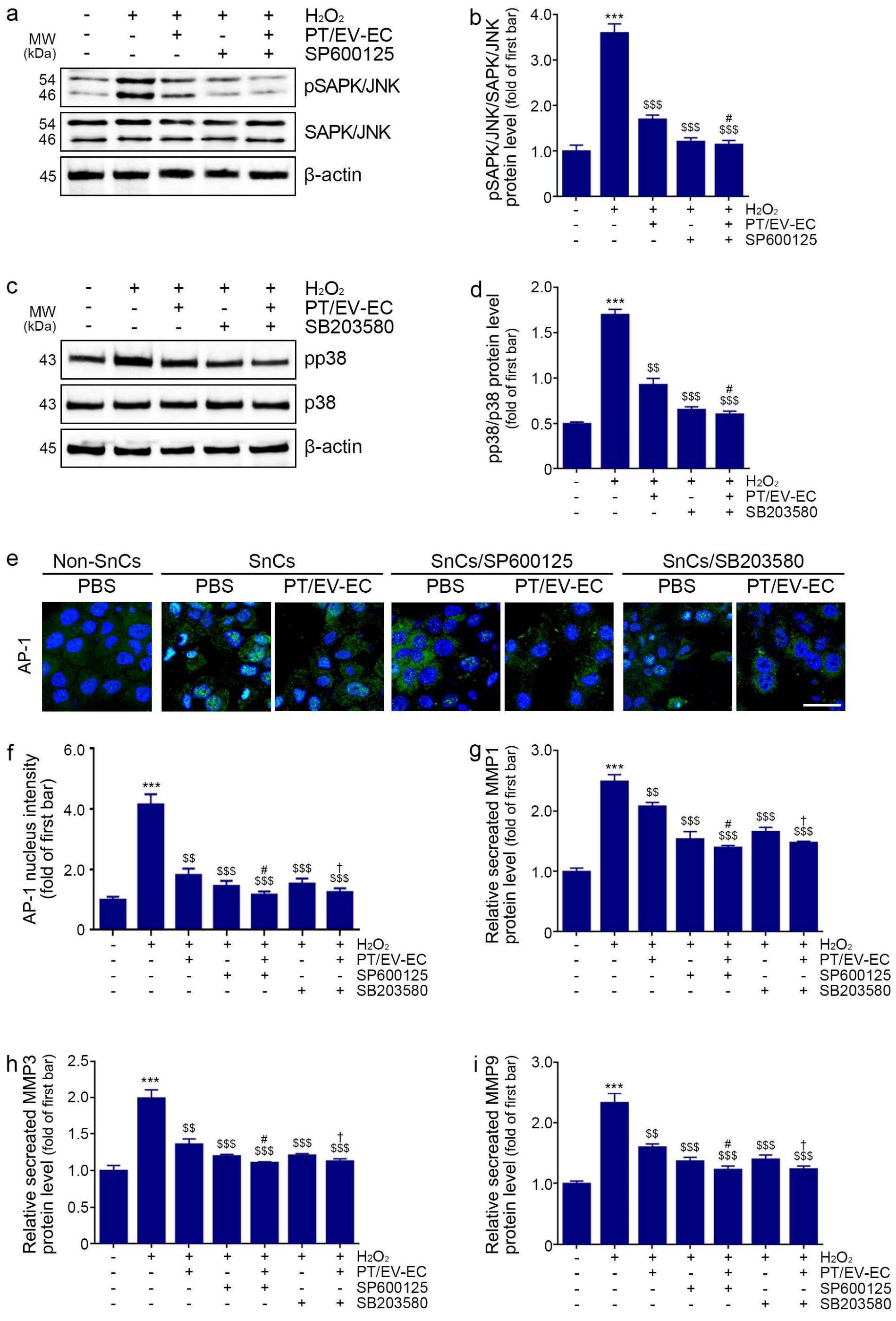

Figure S8. Induction of inhibition of SAPK/JNK and p38 in SnCs keratinocytes treated with EV-EC and PT. (**a, b**) Western blot analysis of pSAPK/JNK, total SAPK/JNK, and β-actin levels in Non-SnCs and SnCs keratinocytes treated with PBS, PT/EV-EC, SP600125, or SP600125/PT/EV-EC. (**c, d**) Western blot analysis of pp38, total p38, and β-actin levels in Non-SnCs and SnCs keratinocytes treated with PBS, PT/EV-EC, SB203580, or SB203580/PT/EV-EC. (**e, f**) ICC analysis of AP-1 expression (green) in Non-SnCs and SnCs keratinocytes (nuclei: blue; scale bar = 30 μm). (**g–i**) ELISA evaluation of MMP1, MMP3, and MMP9 protein levels in Non-SnCs and SnCs keratinocytes. Data are presented as mean ± SD of three independent experiments. ***, $p < 0.001$, first bar vs. second bar; $$, $p < 0.01$ and $$$, $p < 0.001$, second bar vs. third, fourth, fifth, sixth, seventh bars; #, $p < 0.05$, fifth bar vs. fourth bars; †, $p < 0.05$, seventh bar vs. sixth bars (Mann–Whitney U test). ELISA, enzyme-linked immunosorbent assay; ICC, immunocytochemistry; MMP, matrix metalloproteinase; MW, molecular weight.

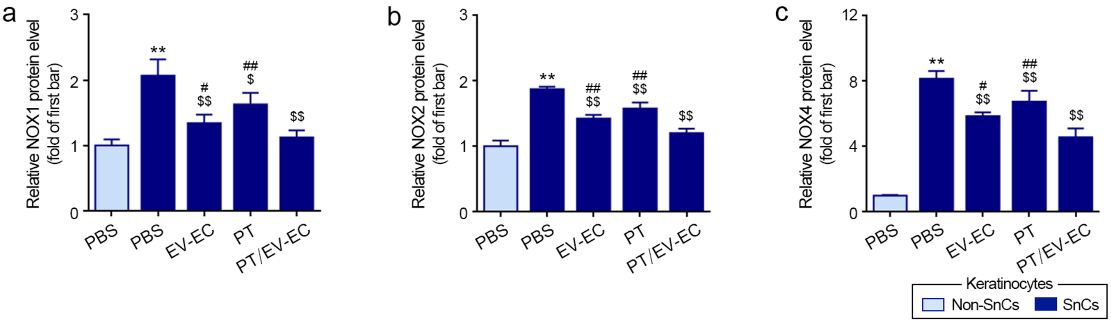

Figure S9. Regulation of NOX1, NOX2, and NOX4 in SnCs keratinocytes treated with EV-EC and PT. (**a, b**) Quantitative assessment of the Western blot data presented in Figure 3a. Data are presented as mean ± SD of three independent experiments. **, $p < 0.01$, first bar vs. second bar; $, $p <$ 0.05 and $$, p < 0.01, second bar vs. third, fourth, and fifth bars; #, $p < 0.05$ and ##, $p < 0.01$ fourth bar vs. third or fifth bars (Mann–Whitney U test). NOX, nicotinamide adenine dinucleotide phosphate oxidase.

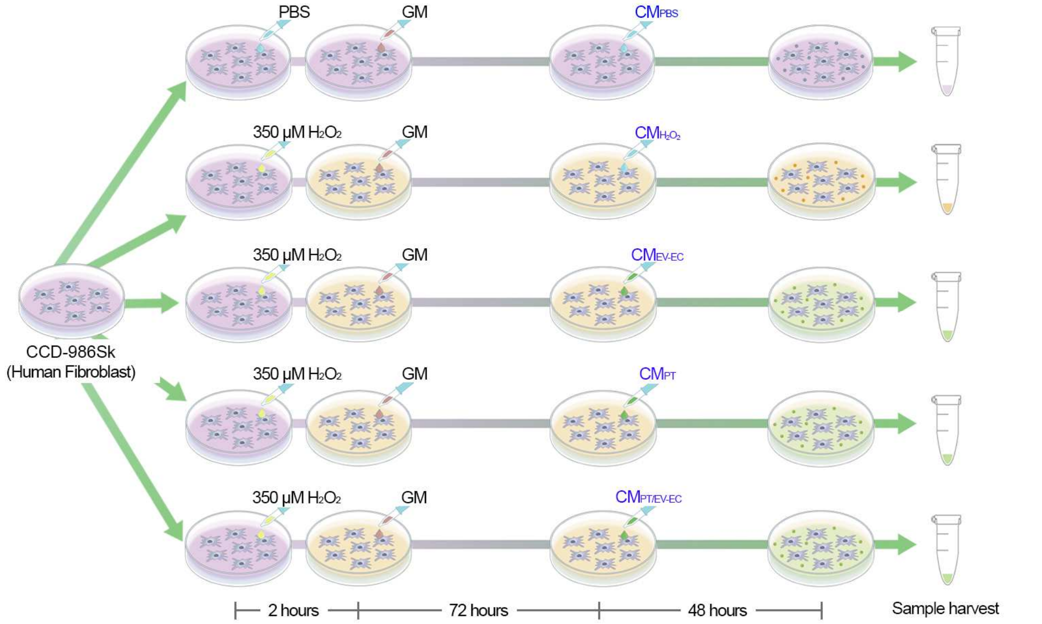

Figure S10. Schematic diagram of the *in vitro* model for fibroblasts exposed to CM from SnCs keratinocytes treated with EV-EC, PT, or PT/EV-EC. Fibroblasts (CCD-986Sk) were exposed to PBS as a control (Non-SnCs(CM$_{PBS}$) group). Cells were treated with 350 μM H_2O_2 for 1.5 h to induce senescence and GM was replaced for 72 h. SnCs fibroblasts were then treated for 48 h with CM consisting of the supernatant from treated keratinocytes as depicted in Figure S4. Cell lysates were collected for further analysis.

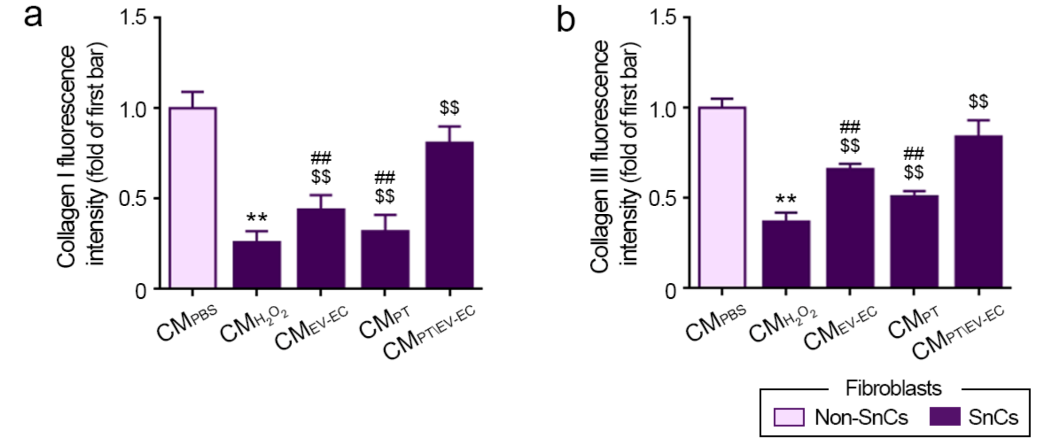

Figure S11. Upregulation of collagen I and III in SnCs fibroblasts exposed to EV-EC- and PT-treated SnCs keratinocyte CM. (a, b) Quantification of the intensity of the cytosolic green signals in Figure 3f. Data are presented as the mean ± SD of three independent experiments. **, $p < 0.01$, first bar vs. second bar; $$, $p < 0.01$, second bar vs. third, fourth, and fifth bars; ##, $p < 0.01$ fifth bar vs. third or fourth bars (Mann–Whitney U test).

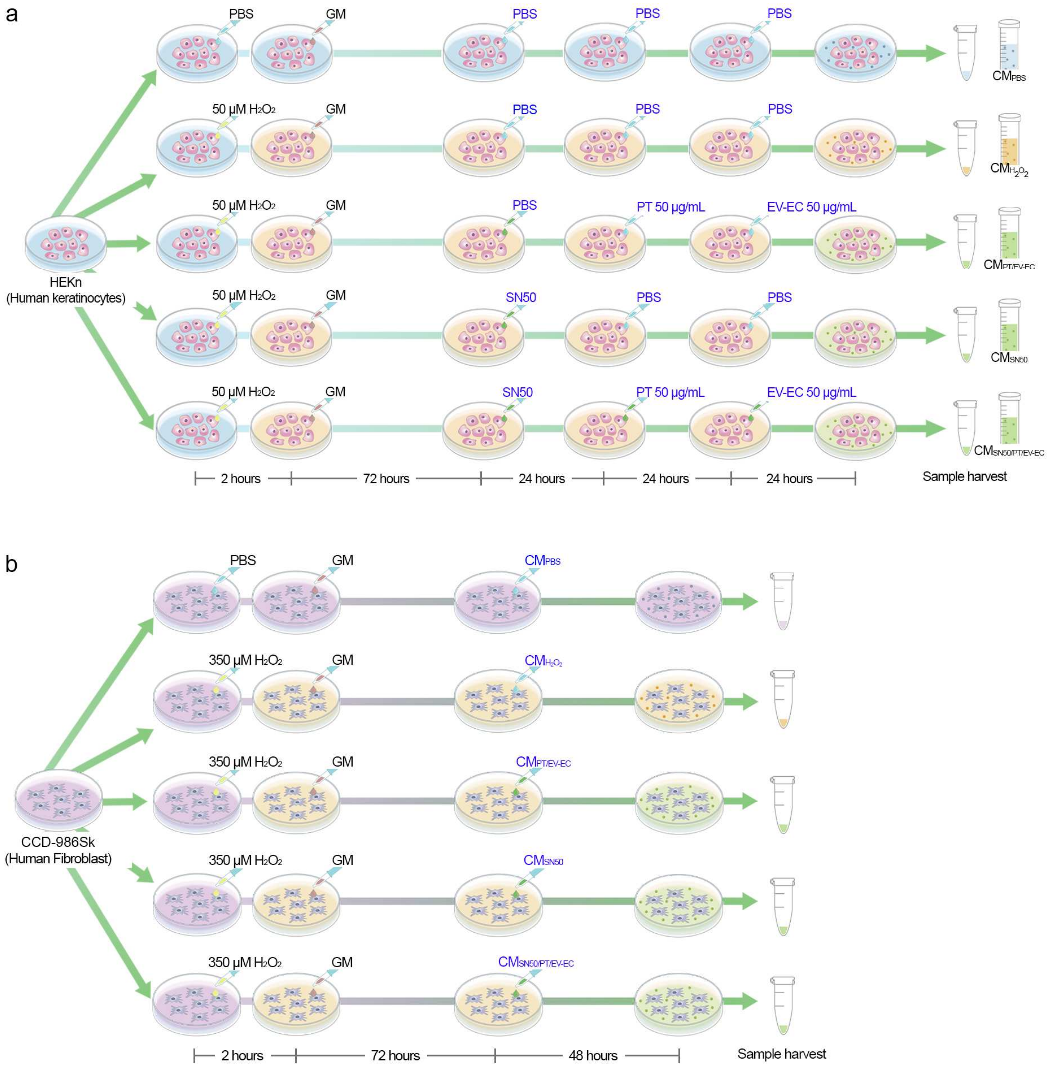

Figure S12. Schematic diagram of the *in vitro* model for keratinocytes and fibroblasts exposed to CM from SnCs keratinocytes treated with PT/EV-EC or following NF-κB inhibition. (a) Keratinocytes were exposed to PBS as a control (Non-SnCs(PBS) group). Cells were treated with 50 µM H_2O_2 for 2 h to induce senescence and GM was replaced for 72 h. SnCs keratinocytes were treated

with PT/EV-EC in the following groups: (1) SnCs(PBS): SnCs keratinocytes treated with PBS. (2) SnCs(PT/EV-EC): SnCs keratinocytes treated with PBS for 24 h and pre-treated with PT (50 μg/mL) for 24 h and then treated with EV-EC (50 μg/mL) for 24 h. (3) SnCs(SN50): SnCs keratinocytes treated with SN50 (10 μM) for 24 h and pre-treated with PBS for 24 h and then treated with PBS for 24 h. (4) SnCs(SN50/PT/EV-EC): SnCs keratinocytes pre-treated with SN50 for 24 h and pre-treated with PT for 24 h and then treated with EV-EC 24 h. (**b**) Fibroblasts (CCD-986Sk) were exposed to PBS as a control (Non-SnCs(CM_{PBS}) group). Cells were treated with 350 μM H_2O_2 for 1.5 h to induce senescence and GM was replaced for 72 h. SnCs fibroblasts were then treated for 48 h with CM, composed of the supernatant from treated keratinocytes as described in Figure S12a. Cell lysates were collected for further analysis.

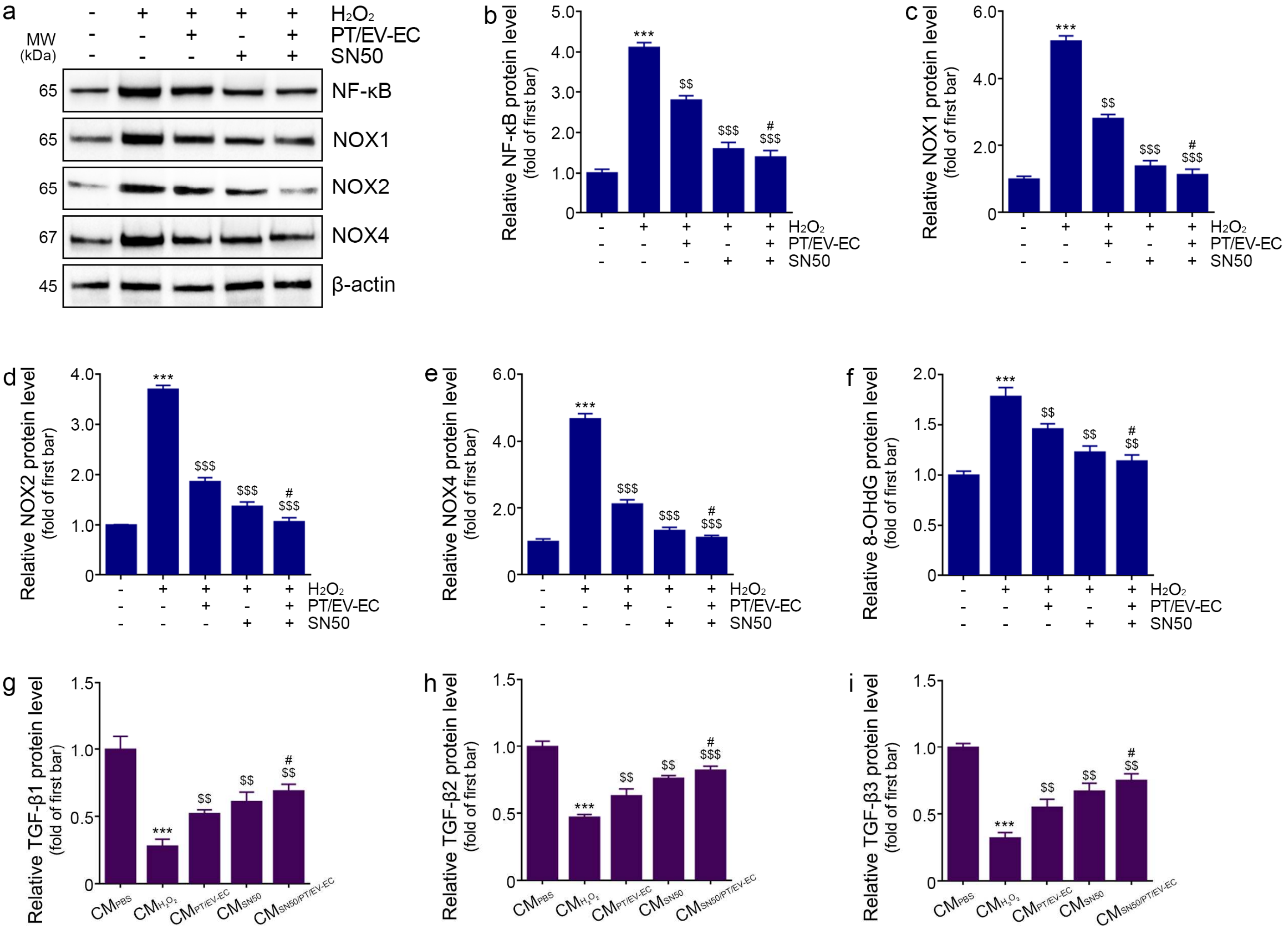

Figure S13. Upregulation of ROS and TGF-β levels in SnCs keratinocytes and SnCs fibroblasts exposed to CM from SnCs keratinocytes treated with PT/EV-EC or following NF-κB inhibition. (**a**-**e**) Western blot analysis of NF-κB, NOX1, NOX2, and NOX4 in Non-SnCs and SnCs keratinocytes treated with PT/EV-EC, SN50, or SN50/PT/EV-EC. (**f**) ELISA evaluation of 8-OHdG protein levels in Non-SnCs and SnCs keratinocytes. (**g–i**) ELISA assessment of TGF-β1, TGF-β2, and TGF-β3 protein levels in Non-SnCs and SnCs fibroblasts treated with conditioned media (CM_{PBS}, $CM_{H_2O_2}$, $CM_{PT/EV\text{-}EC}$, CM_{SN50}, and $CM_{SN50/PT/EV\text{-}EC}$). Data are presented as mean ± SD of three independent experiments. ***, $p < 0.001$, first bar vs. second bar; $$, $p < 0.01$ and $$$, $p < 0.001$, second bar vs. third, fourth, and fifth bars; #, $p < 0.05$, fifth bar vs. third and fourth bars (Mann–Whitney U test). ROS, reactive oxygen species.

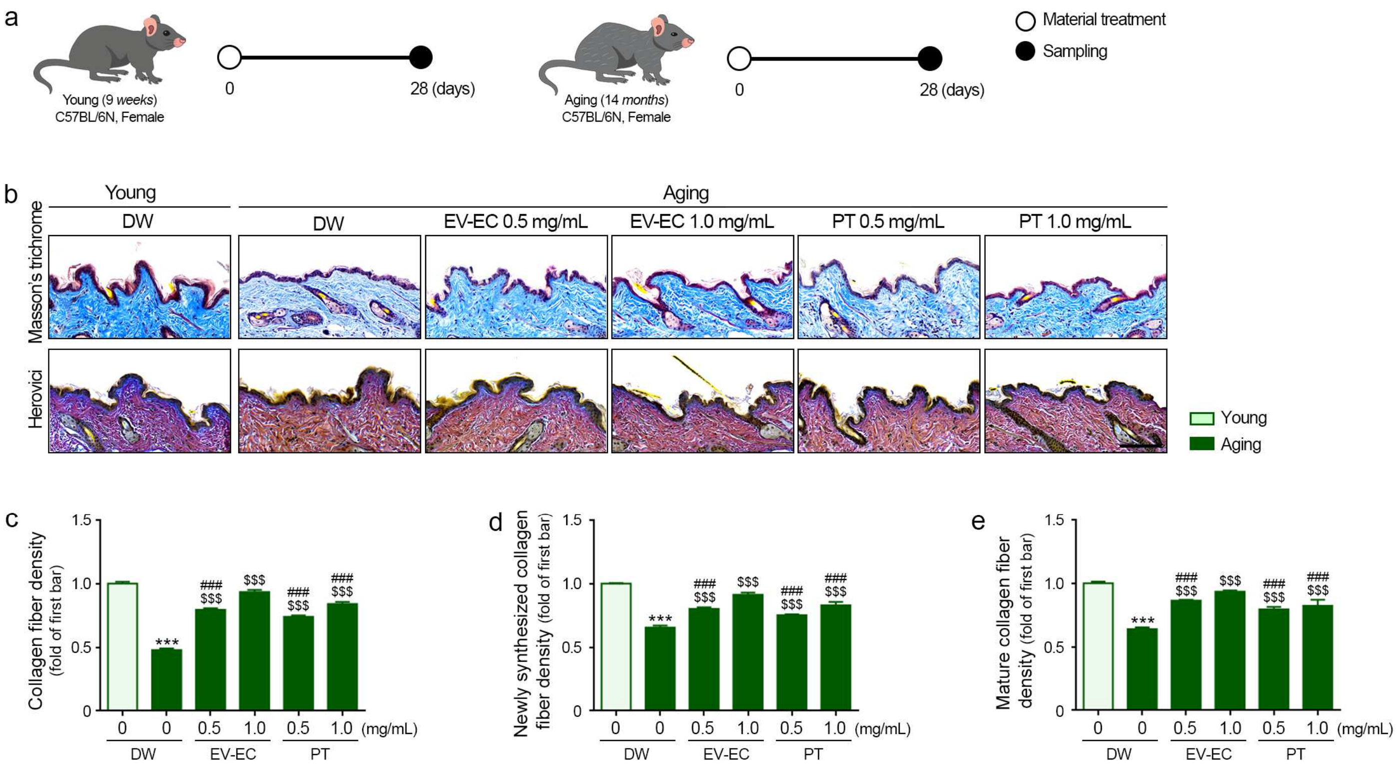

Figure S14. Determination of the optimal treatment dosage of PT and EV-EC in the skin of aged mice. (**a**) Establishment of a mouse (C57BL/6N) model to determine the optimal concentration of PT and EV-EC for promoting collagen synthesis. The back skin of young (9 weeks) and aged mice (14 months) was treated with 0.5 or 1 mg/mL of PT or 0.5 or 1 mg/mL of EV-EC in 200 μL of DW using a MTS. Skin assessment was conducted after 28 days. (**b**) Evaluation of collagen fiber content using Masson's trichrome staining, with representative images shown in the upper panels. Herovici's staining distinguished newly synthesized (blue) and mature (red) collagen fibers in the lower panels (scale bar = 100 μm). (**c–e**) Quantification of collagen fiber density (c; upper panels in b), newly synthesized collagen (d), and mature collagen (e; lower panel in b). Data are presented as mean ± SD of three independent experiments. ***, $p < 0.001$, first bar vs. second bar; $$$, $p < 0.001$, second bar vs. third, fourth, and fifth bars; ###, $p < 0.001$, fourth bar vs. third or fifth bars (Mann–Whitney U test). DW, distilled water; MTS, microneedling system.

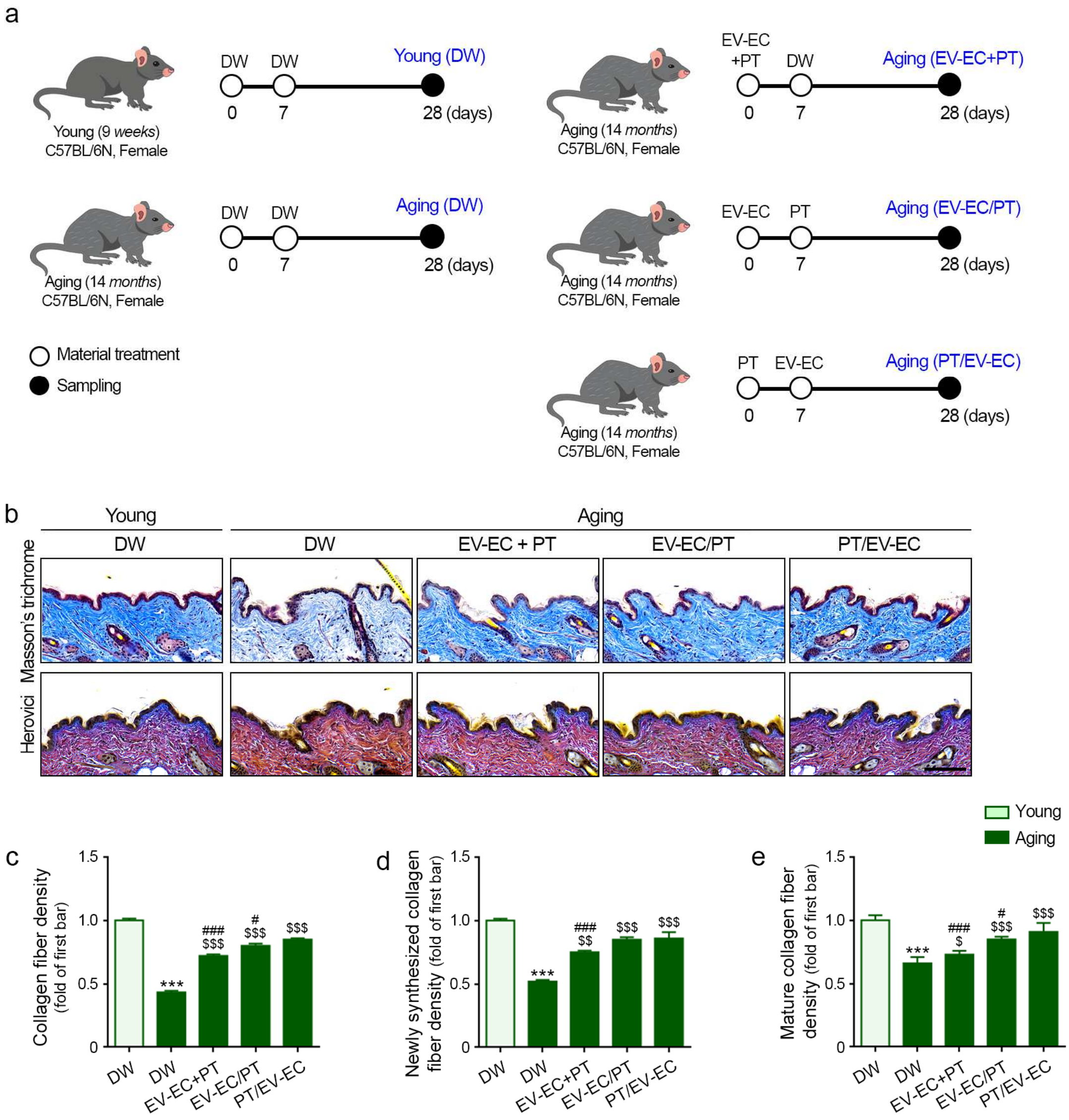

Figure S15. Determination of the optimal EV-EC and PT treatment order in the skin of aged mice. (**a**) Experimental design of the mouse model for evaluating the order of EV-EC and PT treatments to maximize collagen synthesis effects. All treatments were applied via a MTS. Mouse (C57BL/6N) groups were administered 200 µL as follows: Young (9 weeks) and aged mice (14 months) were injected with 200 µL of DW as a control. Aged mice were subjected to three treatment protocols: (1) simultaneous injection with PT and EV-EC, with a DW injection 7 days later (PT+EV-EC), (2) injection with EV-EC, with a PT injection 7 days later (EV-EC/PT), and (3) injection with PT, with an EV-EC injection 7 days later (PT/EV-EC). (**b**) Evaluation of collagen fiber content using Masson's trichrome staining, with representative images shown in the upper panels. Herovici's staining distinguished newly synthesized (blue) and mature (red) collagen fibers in the lower panels (scale bar = 100 µm). (**c**–**e**) Quantification of collagen fiber density (c; upper panels in b), newly synthesized collagen (d), and mature collagen (e; lower panel in b). Data are presented as mean ± SD of three independent experiments. ***, $p < 0.001$, first bar vs. second bar; \$, $p < 0.05$, \$\$, $p < 0.01$ and \$\$\$, $p < 0.001$, second bar vs. third, fourth, and fifth bars; #, $p < 0.05$ and ###, $p < 0.001$, fourth bar vs. third or fifth bars (Mann–Whitney U test).

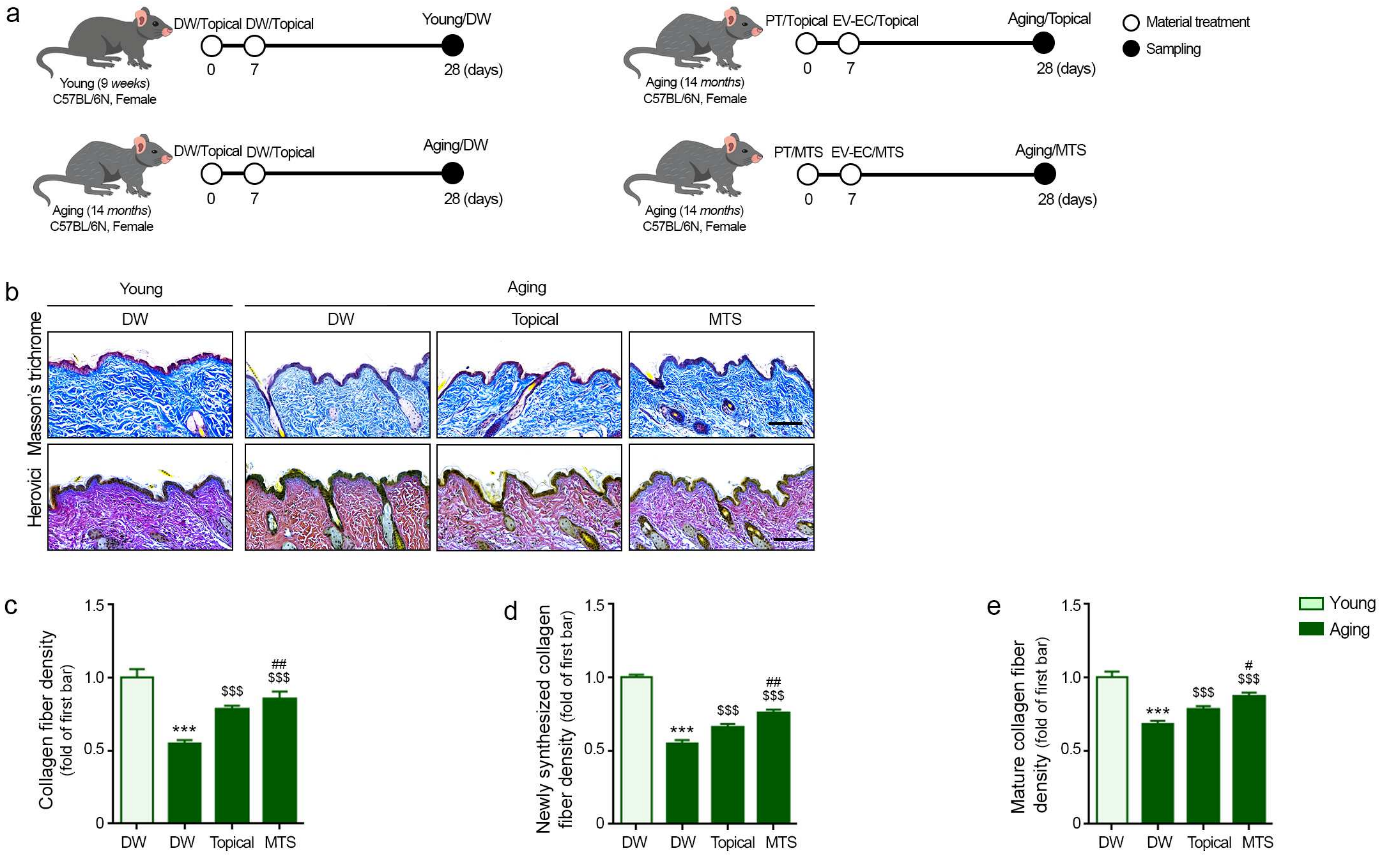

Figure S16. Determination of the optimal application method of topical or MTS treatment in the skin of aged mice. (**a**) Experimental design of the mouse model for evaluating the order of topical or MTS treatment to maximize collagen synthesis effects. Mouse (C57BL/6N) groups were administered 200 µL of total substance, as follows: Young (9 weeks) and aged (14 months) mice were injected with 200 µL of DW as a control. Aged mice were subjected to two treatment protocols: (1) Topical treatment with PT, with EV-EC topical treatment 7 days later (Aging/Topical), and (2) MTS treatment with PT, with EV-EC injection 7 days later (Aging/MTS) (**b**) Evaluation of collagen fiber content using Masson's trichrome staining, with representative images shown in the upper panels. Herovici's staining distinguished newly synthesized (blue) and mature (red) collagen fibers in the lower panels (scale bar = 100 µm). (**c–e**) Quantification of collagen fiber density (c; upper panels in b) and newly synthesized (d) and mature collagen (e; lower panel in b). Data are presented as mean ± SD of three independent experiments. ***, $p < 0.001$, first bar vs. second bar; $$$, $p < 0.001$, second bar vs. third and fourth bars; #, $p < 0.05$ and ##, $p < 0.01$, fourth bar vs. third bar (Mann–Whitney U test).

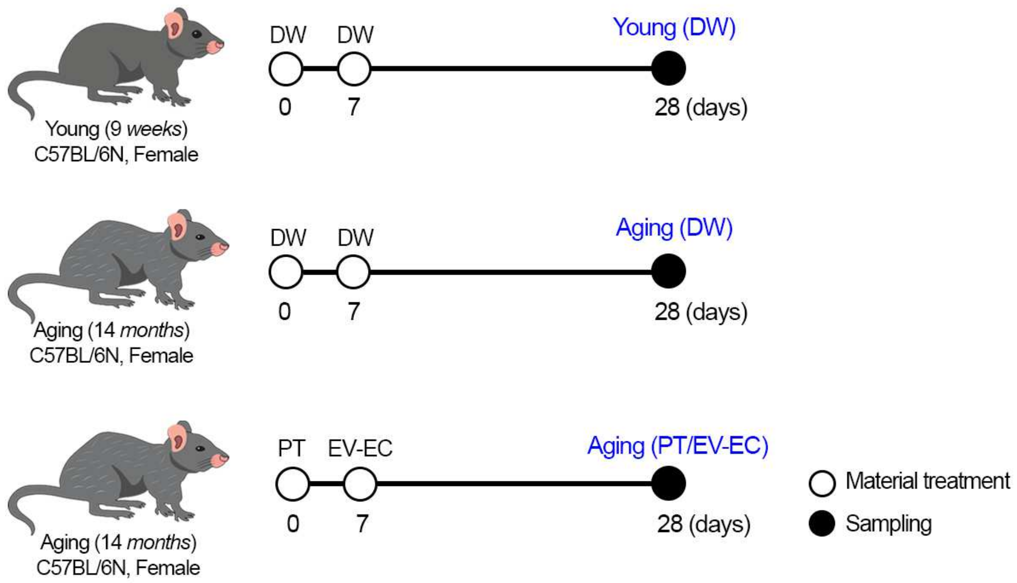

Figure S17. Mouse model for efficacy verification of EV-EC and PT treatment in skin rejuvenation. The back skin of young (9 weeks) and aged (14 months) mice was injected via a MTS with either DW or 1 mg/mL PT (200 μL). After 7 days, either DW or 1 mg/mL of EV-EC (200 μL) was injected. Skin assessment was conducted 28 days after the first injection. DW injections into young mice were used as a control.

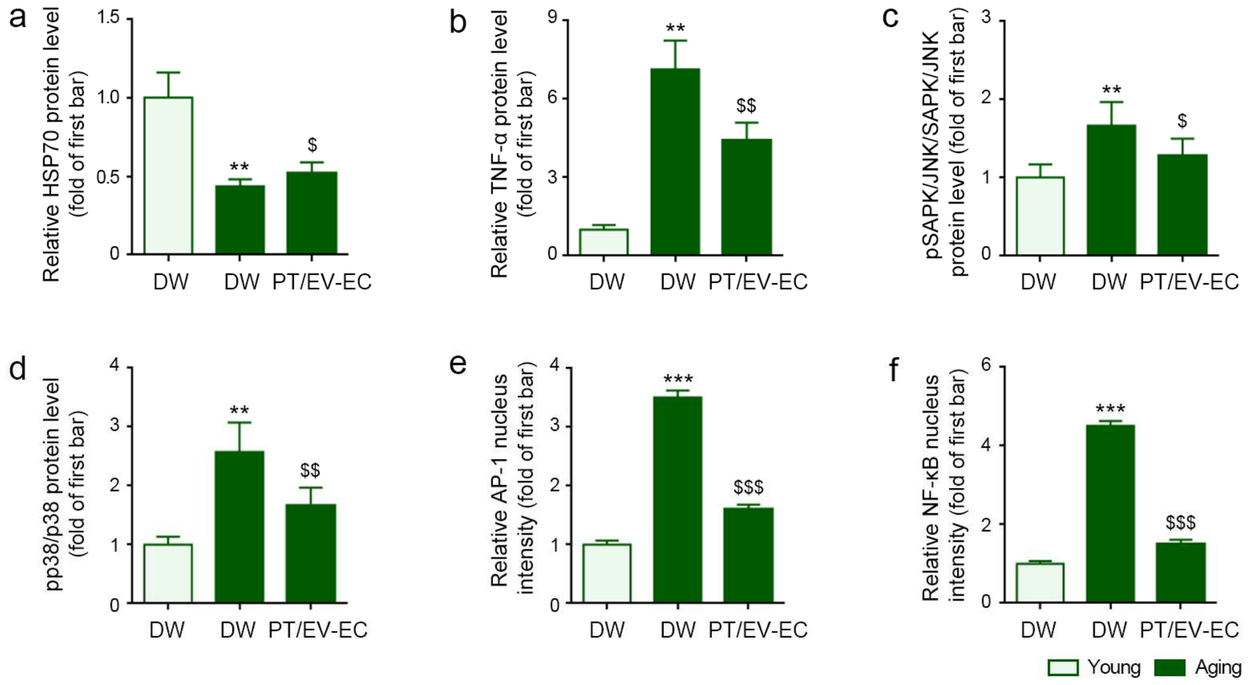

Figure S18. Regulation of HSP70, TNF-α, MAPK, AP-1, and NF-κB expression in the skin of the aged mice treated with PT/EV-EC. (**a, b**) Quantitative assessment of the Western blot data presented in Figure 4a. (**c, d**) Quantitative assessment of the Western blot data presented in Figure 4b. (**e, f**) Quantification of the nuclear brown signal intensity in Figure 4c. Data are presented as mean ± SD of three independent experiments. **, $p < 0.01$ and ***, $p < 0.001$, first bar vs. second bar; $, $p <$ 0.05, $$, $p < 0.01$ and $$$, $p < 0.001$, second bar vs. third bar (Mann–Whitney U test).

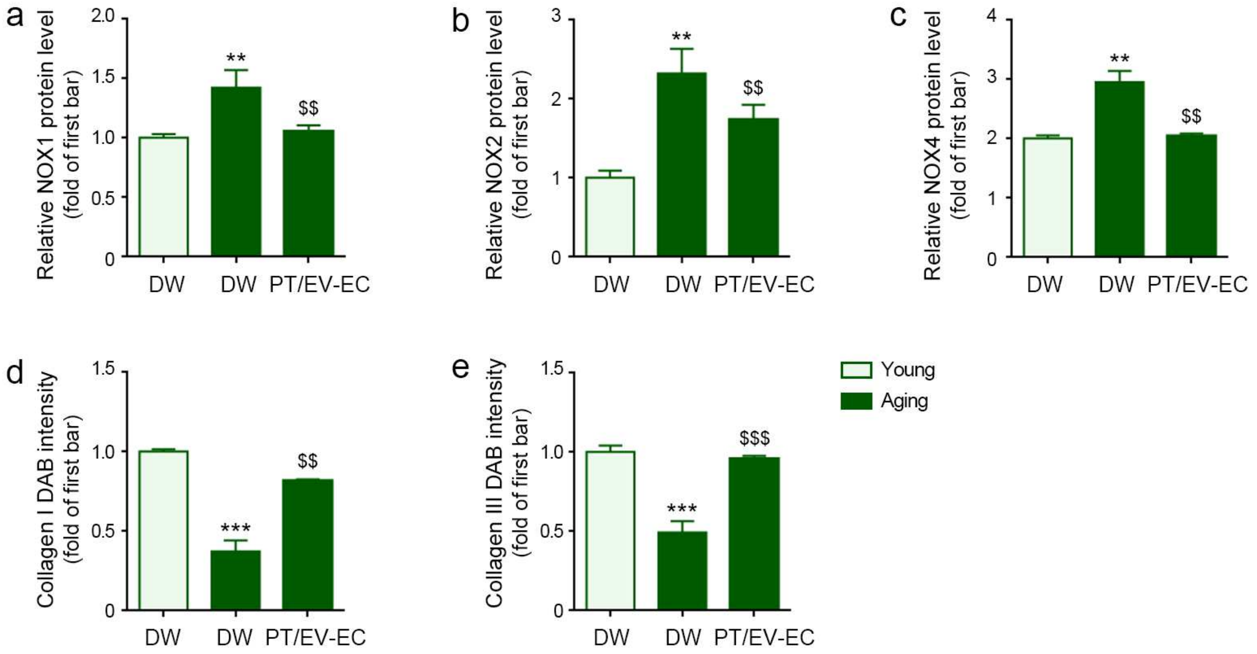

Figure S19. Regulation of NOX1, NOX2, NOX4, collagen I, and collagen III levels in the skin of aged mice treated with PT/EV-EC. (**a–c**) Quantitative assessment of the Western blot data presented in Figure 5a. (**d, e**) Quantification of the dermal brown signal intensity in Figure 5f. Data are presented as mean ± SD of three independent experiments. **, $p < 0.01$ and ***, $p < 0.001$, first bar vs. second bar; $$, $p < 0.01$ and $$$, $p < 0.001$, second bar vs. third bar (Mann–Whitney U test).

Table S1. Dynamic light scattering analysis and nanoparticle-tracking analysis of the EV-EC utilized in this study.

Dynamic light scattering	Average diameter	153.0 ± 39.9 nm
Nanoparticle-tracking analysis	Average diameter	137.6 ± 4.7 nm
	Mode diameter	81.5 ± 3.1 nm
	Dilution concentration	$2.21 \times 10^{8} \pm 3.84 \times 10^{7}$ particles/mL (2,000×)
	Concentration per 1 g *E. cava*	$3.68 \times 10^{10} \pm 6.40 \times 10^{9}$ particles/mL

Data represent the mean ± SD of three independent experiments. EV-EC, extracellular vesicles from *Ecklonia cava*.

Table S2. List of overexpression and inhibitors.

Name		Company	Catalog No.	Dilution rate
HSP70 overexpression	GGA	MedChemExpress	HY-B0779	30 μM
JNK inhibitor	SP600125	Sigma-Aldrich	S5567	50 μM
NF-κB inhibitor	SN50	Santa Cruz Biotechnology	SC3060	10 μM
p38 inhibitor	SB 203580	Sigma-Aldrich	559389	20 μM

Table S3. Primers utilized for quantitative reverse transcription-polymerase chain reaction.

Gene		Primers
Actb	Forward	5'-GGG ACC TGA CTG ACT ACC TCA T-3′
	Reverse	5'-CCT TAA TGT CAC GCA CGA TTT-3′
HSP70	Forward	5'-TGA GGG TAA GAT GAT CAT GCA G-3′
	Reverse	5'-ACA TAT TCC TCC ACT GCG TTC T-3′
P16	Forward	5'-ACC AGA GGC AGT AAC CAT GC-3′
	Reverse	5'-TGC TTC TAC AAA CCC ACA AAT G-3′
P21	Forward	5'-GGA GAC TCT CAG GGT CGA AAA-3′
	Reverse	5'-GCT TCC TCT TGG AGA AGA TCA G-3′
TNF-α	Forward	5'-CTC TCT CTA ATC AGC CCT CTG G-3′
	Reverse	5'-GTT TGC TAC AAC ATG GGC TAC A-3′

Table S4. Antibodies utilized for Western blot, ELISA, ICC, and IHC.

Antibody	Company	Catalog No.	Dilution rate			
			ELISA	Western blot	ICC	IHC
8-OHdG	GeneTex	GTX41980	1:500			
AP-1	LifeSpan BioSciences	LS-C826302			1:50	1:100
β-actin	Cell Signaling Technology	4967		1:1,000		
Collagen I	Santa Cruz Biotechnology	sc-293182			1:50	1:50
Collagen III	Bioss Antibodies	bs-0549R			1:100	1:200
HSP70	StressMarq Biosciences	SPC-103		1:1,000		
Laminin	Novus Biologicals	NBP300-144				1:200
MMP1	FineTest	FNab05233	1:200			
MMP3	ABclonal	A1202	1:1,000			
MMP9	ABclonal	A0289	1:1,000			
NF-κB	Cell signaling Technology	8242		1:1,000	1:200	1:200
Nidogen	Santa Cruz Biotechnology	sc-47773				1:50
NOX1	Affinity Biosciences	DF8684		1:1,000		
NOX2	Affinity Biosciences	DF6520		1:1,000		
NOX4	Affinity Biosciences	DF6924		1:1,000		
p38	Cell signaling Technology	9212		1:1,000		
pp38	Cell signaling Technology	4511		1:1,000		
pSAPK/JNK	Cell signaling Technology	9251		1:1,000		
SAPK/JNK	Cell signaling Technology	9252		1:1,000		
TGF-β1	StressMarq Bioscience	SPC-1312	1:2,000			
TGF-β2	FineTest	FNab08639	1:1,000			

TGF-β3	FineTest	FNab08640	1:1,000			
TNF-α	Novus Biologicals	nbp1-19532		1:500		

AP-1, activator protein 1; β-actin, beta-actin; ELISA, Enzyme-linked immunosorbent assay; HSP70, heat shock protein 70; ICC, Immunocytochemistry; IHC, Immunohistochemistry; MMP, matrix metalloproteinases; NF-κB, nuclear factor-kappa B; NOX, NADPH oxidases; pp38, phosphorylated p38; pSAPK/JNK, phosphorylated SAPK/JNK; SAPK/JNK, stress-activated protein kinases/jun amino-terminal kinases; TGF-β, transforming growth factor-beta; TNF-α, tumor necrosis factor-alpha; 8-OHdG, 8-hydroxy-2′-deoxyguanosine.
